# Controlling Release Kinetics of an Adjuvant from a Depot Improves the Efficacy of Local Immunotherapy in Metastatic Cancer

**DOI:** 10.1002/advs.202503591

**Published:** 2025-07-06

**Authors:** Joshua J. Milligan, Rachel L. Strader, Arunangshu Chakrabarty, Max Ney, Parul Sirohi, Jonathan C. Su, Anastasia K. Varanko, Yulia Shmidov, Kalina Tsolova, Cassio Mendes Fontes, Anna Finkelstein, Rohinee Mattikalli, Yun‐Xing Wang, Lixin Fan, Xinghai Li, Smita K. Nair, Ashutosh Chilkoti

**Affiliations:** ^1^ Department of Biomedical Engineering Duke University Durham NC 27708 USA; ^2^ Department of Biochemistry Duke University School of Medicine Durham NC 27708 USA; ^3^ Center for Structural Biology Center for Cancer Research National Cancer Institute Fredrick MD 21702 USA; ^4^ Basic Science Program Fredrick National Laboratory for Cancer Research SAXS Facility of the National Cancer Institute Fredrick MD 21702 USA; ^5^ Department of Surgery Duke University Medical Center Durham NC 27708 USA

**Keywords:** biopolymers, drug delivery, elastin‐like polypeptides, immunotherapy

## Abstract

Biomaterials can improve cancer immunotherapies by controlling their release and thereby optimizing their time‐dependent engagement of the immune system. In this study, an approach is described to control the release of a potent immunostimulant—CpG oligodeoxynucleotide—from a genetically‐encoded elastin‐like polypeptide (ELP) depot. A CpG‐binding ELP containing an oligolysine domain (ELP‐Lys_12_) is synthesized that electrostatically complexes CpG and formulate it with an excipient ELP. The ELP‐CpG complex retains the thermally responsive phase behavior of the parent ELP, transitioning into a viscous depot at body temperature. Stepwise addition of excipient ELP predictably changes ELP‐CpG transition temperature, depot dissolution kinetics, and retention of CpG within the depot. Mixtures of ELP‐Lys_12_, excipient ELP, and CpG undergo microphase separation, forming a porous, sponge‐like depot that contains tunable amounts of soluble CpG in the pores. In vivo, the modified formulations exhibit varying degrees of CpG retention over multiple weeks following a single intratumoral injection. Finally, by modifying the release kinetics of CpG, optimized ELP‐CpG achieves greater reduction of metastatic disease in a murine metastatic breast cancer model than soluble CpG. These results demonstrate that ELPs can be used to precisely tune the release kinetics of immunotherapies for better outcomes in the treatment of metastatic cancer.

## Graphical abstract

We show that intratumoral release of an immunotherapy—CpG —can be tuned with molecular precision by complexing it to an elastin‐like polypeptide (ELP). Injecting ELP‐CpG and an excipient ELP into tumors drives thermally‐triggered phase separation into a micro‐scale “sponge” depot that releases CpG at rates depending on the ELP composition, which correlates with control of metastatic breast cancer in mice.

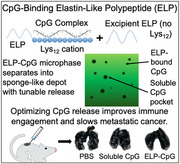

## 1 Introduction

Metastatic cancer is a tremendous public health challenge, being responsible for the majority of all cancer deaths.^[^ ^1^ ^]^ While interventions such as early detection, surgery, chemotherapy, and radiotherapy have greatly improved outcomes for patients with local disease, patients with metastatic disease do not usually survive.^[^ ^2^ ^]^ Immunotherapy has great potential to treat these patients by training the immune system to recognize and attack distant metastases that are otherwise poorly detectable or untreatable.^[^ ^3^ ^]^ Despite its promise, the clinical use of immunotherapy remains limited to hematological and other non‐solid tumors because of the myriad of delivery challenges to solid tumors, such as their high interstitial fluid pressure, hypoxia‐induced necrosis of the tumor core, and tumor heterogeneity.^[^ ^4^ ^]^

Controlled release biomaterials are a promising new approach to deliver immunotherapy to solid tumors. While many cancer drugs are poorly effective on their own because of their short half‐life, low and heterogeneous intratumoral (*i.t*.) uptake, or dose‐limiting toxicity,^[^ ^5^ ^]^ controlled‐release formulations can address these shortcomings.^[^ ^6^ ^]^ One immunotherapeutic agent whose efficacy has been improved through controlled‐release platforms is CpG,^[^ ^7^ ^]^ an immunostimulatory repeat of unmethylated cytosine and guanine nucleotides first identified as a bacterial pathogen‐associated molecular pattern. Due to its immunogenicity, CpG is used as an adjuvant in cancer immunotherapy and vaccines.^[^ ^8^ ^]^ CpG oligodeoxynucleotide 1826 (CpG ODN 1826) is a 20‐base, 6.3 kDa DNA that strongly binds to toll‐like receptor 9 (TLR9) in mammalian plasmacytoid dendritic cells (pDCs).^[^ ^9^ ^]^ This activates NF‐κB‐mediated production of type I interferons, prompting DC uptake of antigenic material and presentation to naïve T cells.^[^ ^10^ ^]^ CpG has exhibited mediocre efficacy in numerous trials, however, due to dose‐limiting reactions and poor tumor‐specific immunogenicity.^[^ ^11^ ^]^ Locally delivered, sustained release formulations of CpG have been proposed to improve DC activation and neoantigen uptake within tumors while minimizing systemic toxicity.^[^ ^12^ ^]^ Further, there is also evidence that CpG formulations exhibiting local aggregation or higher‐order assembly support greater intracellular uptake and, therefore, immunogenicity.^[^ ^13^ ^]^

We previously described a strategy to achieve a long‐lasting CpG formulation using an elastin‐like polypeptide (ELP) carrier.^[^ ^14^ ^]^ ELPs are synthetic intrinsically disordered proteins that consist of repeats of the pentapeptide motif [Val‐Pro‐Gly‐Xaa‐Gly]_n_, where Xaa is any amino acid except Pro.^[^ ^15^ ^]^ ELPs undergo liquid‐liquid phase separation via a lower critical solution temperature (LCST) phase transition.^[^ ^15^ ^]^ At the transition temperature (T_t_), the ELP forms an insoluble coacervate phase, and the T_t_ of the ELP can be tuned by the choice of guest residue and molecular weight to yield ELPs that will form a depot upon injection in vivo.^[^ ^16^ ^]^ In previous work, we reported the *i.t*. delivery of CpG electrostatically complexed to an ELP with a positively charged, 12‐mer oligolysine tail (ELP‐Lys_12_) that bound to the negatively charged phosphate backbone of CpG, slowly releasing CpG over 2–3 weeks following injection.^[^ ^14^ ^]^ This original, unoptimized ELP‐CpG formulation was combined with *i.t*. brachytherapy, and the combination therapy synergistically inhibited local tumor growth while also significantly reducing metastatic burden in a murine model of triple‐negative breast cancer.

To optimize the release of the CpG from the depot, herein we explore two complementary strategies: 1) control of the affinity of the ELP for CpG by systematically varying the number of Lys residues in the ELP‐Lys_n_ fusion; and 2) modulating the rebinding kinetics of CpG to ELP‐Lys_12_ by doping in excipient ELP that lacks the CpG‐binding oligolysine domain. We find that varying the number of Lys residues from 4–12 has no effect on tumor growth. In contrast, we find that adjusting the ratio of ELP‐Lys_12_ to excipient ELP creates a ternary system that—surprisingly—leads to microphase separation of the CpG into two populations: a population that is complexed to the ELP‐Lys_12_ and free CpG that exists as pockets embedded in a matrix of co‐existing ELP‐Lys_12_ and excipient ELP phases. We demonstrate that altering the ratio of the two ELPs in the depot modulates the ratios of CpG in the two—bound versus free—populations and thereby tunes the release kinetics of CpG from the depot. We identify an optimized formulation that significantly reduces lung metastases in the 4T1 mammary carcinoma model compared to soluble CpG and other ELP‐CpG formulations. These results suggest that systematically optimizing a mixed depot of ELP‐oligolysine and an excipient ELP is a viable strategy to maximize the therapeutic effect of an adjuvant for *i.t*. delivery.

## 2 Results

We previously showed that CpG electrostatically complexed with ELP‐Lys_12_ enhances internalization of CpG into the early endosomal compartment of dendritic cells (DCs).^[^ ^14^ ^]^ Upon *i.t*. injection, the ELP‐oligolysine/CpG complex undergoes an LCST phase transition at body temperature to create a coacervate phase that functions as a long‐lasting depot of CpG.

### 2.1 Varying the Affinity of CpG for ELP‐Oligolysine

Initially, we tried to control CpG release from an *i.t*. depot by reducing the number of positively charged Lys residues within the ELP‐Lys_12_ fusion. We chose stepwise, 4‐mer reductions in the number of Lys residues by producing fusions of ELP to Lys_4_ and Lys_8_ in addition to the original ELP‐Lys_12_. After expressing, purifying, and characterizing the ELP‐Lys_4_ and ‐Lys_8_ fusions as described previously (Figure S1, Supporting Information), we evaluated the thermally responsive phase behavior of the fusions with and without CpG mixed with ELP‐Lys_n_ at a 1:1 ratio of positively charged Lys amine groups to negatively charged DNA phosphate groups (N:P ratio, Figure S2, Supporting Information). Consistent with our previous work,^[^ ^14^ ^]^ at a biologically relevant concentration of 1000 µм, the fusions alone had T_t_s of 31.5 °C (ELP‐Lys_4_) and 29.2 °C (ELP‐Lys_8_), which decreased to 28.8 °C and 26.3 °C, respectively, upon the addition of CpG.

Next, we investigated the binding affinity of each ELP‐Lys_n_ construct to CpG using a DNA gel‐shift assay. Briefly, CpG and ELP were mixed at a 1:1 N:P ratio and run on an agarose gel under electrophoresis. When ELP‐Lys_n_ complexes with CpG, the charge‐neutralized DNA no longer migrates through the gel to its expected position based on molecular weight, and its migration is entirely retarded if the charge of the CpG is completely neutralized by the ELP. We clearly observed (Figure S3A, Supporting Information) that migration of ELP‐Lys_12_‐CpG was completely retarded in the gel, indicating that the ELP‐Lys_12_ completely neutralizes the charge of the CpG. In contrast, ELP‐Lys_8_ and ‐Lys_4_, respectively, exhibit decreasing fractions of charge‐neutralized CpG, demonstrating that even at an equal N:P ratio between ELP and CpG for all three constructs, ELP‐Lys_4_ and ‐Lys_8_ bind CpG to a lesser extent than ELP‐Lys_12_. We confirmed these findings using surface plasmon resonance (SPR), in which we immobilized a CpG‐biotin conjugate onto a polyethylene glycol (PEG)‐functionalized chip and flowed ELP‐Lys_4, 8, or 12_ over the chip. We measured the association and dissociation binding kinetics between the ELPs and CpG, and analyzed them using a steady‐state binding model (Figure S3B–D, Supporting Information). We found that ELP‐Lys_12_ binding with CpG exhibits a dissociation constant (K_D_) of ≈1.6 × 10^−6^ м, which was approximately three times stronger than that of ELP‐Lys_4_ and ‐Lys_8_, which exhibited similar K_D_ values of ≈5.3–5.5 × 10^−6^ м.

As we describe in a prior study,^[^ ^14^ ^]^ we proceeded to investigate CpG complexed with ELP‐Lys_4_ and ELP‐Lys_8_ in a combination therapy with ELP “liquid brachytherapy” that involves the *i.t*. injection of a radioactive ^131^I‐ELP conjugate into the core of the tumor.^[^ ^14^ ^]^ We inoculated female BALB/c mice (n = 6–8) with orthotopic 4T1 tumors and grew the tumors to ≈100 mm^3^. As described previously,^[^ ^14^ ^]^ mice were treated *i.t*. with ELP‐Lys_n_ (n = 4, 8, and 12) complexed to CpG at a 1:1 N:P ratio, followed 24 h later by a single *i.t*. injection of a ^131^I‐ELP conjugate at a dose of 3.3 µCi mm^−3^ of tumor tissue. Changing the number of Lys residues in the ELP had no impact on tumor volume or survival of the mice (Figure S4, Supporting Information). This was an unexpected result, as we anticipated observing some type of therapeutic effect from changing the binding affinity of the CpG to ELP‐Lys_n_.

In analyzing our data in the context of our experimental design, we determined that the approach of tuning binding affinity by adjusting the number of Lys residues on the ELP is not the ideal way to tune CpG release rate from an in vivo depot. This is because our experiment was designed to maintain an equal charge ratio between the ELP and DNA in all three groups (N:P ratio of 1). To maintain the same N:P ratio for ELP‐Lys_4_ as is the case in ‐Lys_12_, while keeping the same injection volume, the concentration of ELP‐Lys_4_ must be scaled by a factor of 3. By doing so, the depot‐dissolution kinetics of the ELP‐CpG was changed, as ELP depots at a higher concentration generally exhibit longer in vivo retention than those of lower concentrations.^[^ ^15^ ^]^ Because of this, we hypothesize that this is a confounding variable that worked against our goal of releasing CpG faster in the depots with fewer Lys residues, as the depot dissolution would be slower and hold onto CpG potentially even longer than in the original ELP‐Lys_12_ depot. Due to the challenges of in vivo delivery with reducing the number of Lys residues on the ELP, we sought to revise our approach moving forward. Instead of directly tuning binding affinity of the ELP and CpG, we decided to tune the rebinding kinetics by doping the ELP‐CpG formulations with an excipient ELP that lacks the Lys_12_ domain.

### 2.2 Tuning the binding of CpG in an ELP‐CpG depot

Our strategy for delivering CpG as an *i.t*. ELP‐bound depot relied on the modular composition of our ELP‐CpG formulation, which comprises a 1:1 molar ratio of ELP‐Lys_12_ to excipient ELP (an identical polypeptide without the C‐terminal Lys_12_). This excipient ELP was added: 1) to promote depot formation by increasing the overall ELP concentration in the depot; and 2) to accomplish the first aim while maintaining a 1:1 N:P ratio between the ELP‐Lys_12_ and CpG. Now, to tune the binding kinetics of CpG to ELP‐Lys_12_, we decreased the molar concentration of ELP‐Lys_12_ and proportionally increased the concentration of excipient to maintain the same overall ELP concentration. Given the relatively weak (micromolar K_D_) binding interactions of ELP‐Lys_12_ and CpG, we hypothesized that changing the depot composition would significantly affect ELP‐CpG binding, dissociation, and subsequent re‐binding kinetics, affecting CpG retention from within depots of varying compositions.

**Figure 1:**
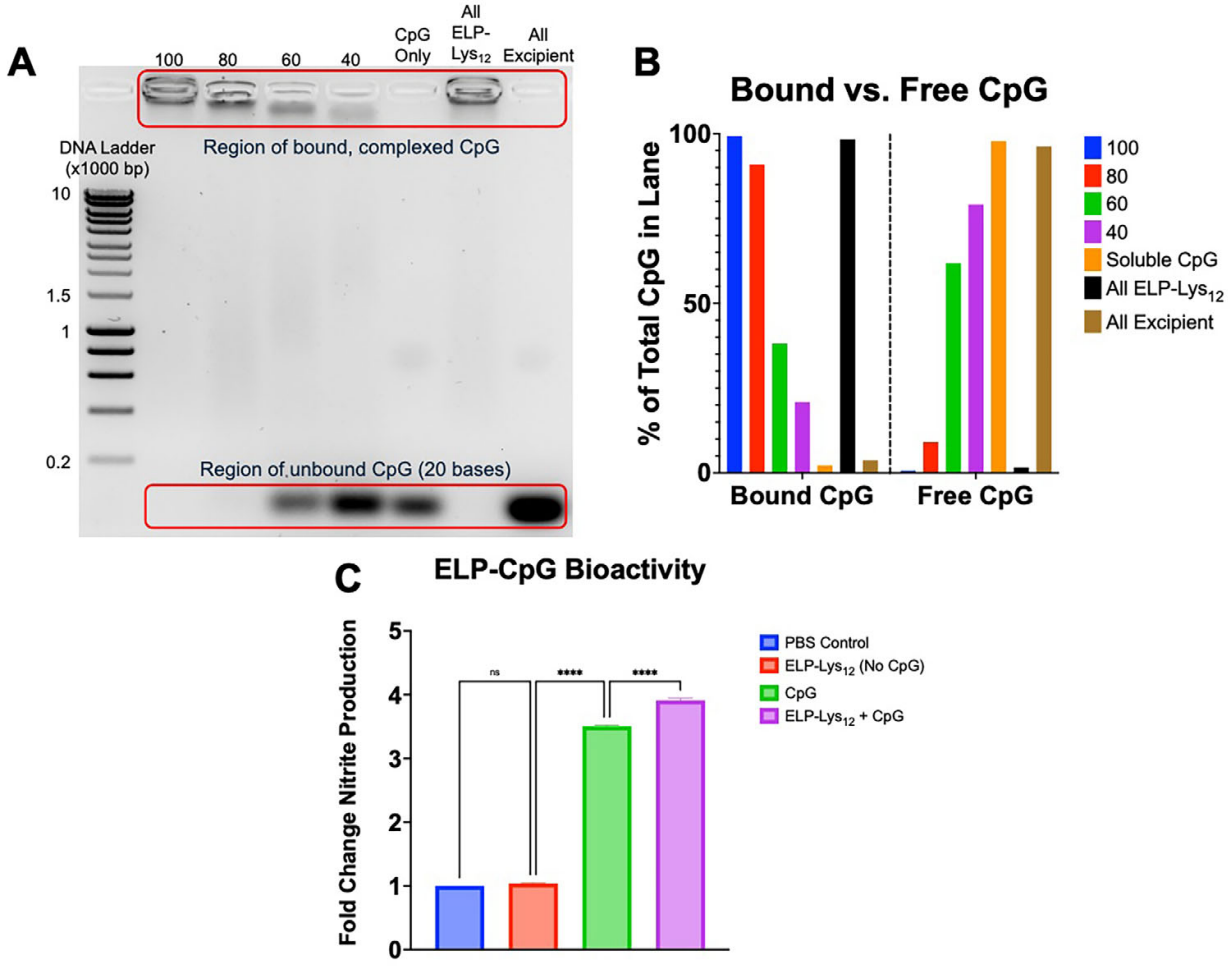
Binding affinity of CpG to ELP‐Lys_12_ is tunable by increasing the proportion of excipient ELP, and the resulting complex is bioactive. A) ELP (60 µм total concentration) was mixed with CpG (18 µм), run in an agarose gel stained with SYBR‐Safe DNA stain, and imaged with a UV transilluminator. Lanes (left‐to‐right) consist of: 10 KB DNA ladder, 100‐, 80‐, 60‐, and 40% ELP‐Lys_12_ as in original formulation, CpG‐only control, all ELP‐Lys_12_ (no excipient), all excipient (no ELP‐Lys_12_). An increased fraction of CpG (20 bp) migrating toward the bottom of the gel is indicative of less ELP‐CpG complexation. B) Quantitative analysis of bound‐ versus unbound‐CpG fraction for each formulation. C) Griess nitrite quantification assay results displaying fold‐change in nitrite production for RAW264.7 macrophages (n = 3 wells per condition) stimulated 24 h with: PBS, ELP‐Lys_12_ alone, CPG, or ELP‐Lys_12_ + CpG at a 1:1 N:P ratio. ns: not significant, ****p < 0.0001 (ANOVA).

We initially screened six formulations (**Table** **1**), the first having the same amount of ELP‐Lys_12_ as in our previous formulation comprising a 1:1 molar ratio of ELP‐Lys_12_ to excipient ELP (100%, or “100”), and the last containing 40% of the ELP‐Lys_12_ as in our original formulation (“40”). Two of the remaining formulations comprised incremental 20% decreases in ELP‐Lys_12_ concentration (80‐, and 60% ELP‐Lys_12_, or “80” and “60”). Finally, two formulations comprised a control of only ELP‐Lys_12_ with no excipient ELP (“All Lys_12_”) and a control of only excipient ELP (“All excipient”). To compare CpG binding in the six formulations, we performed a DNA gel‐shift assay as described previously.^[^ ^14^ ^]^ A CpG‐only control, as well as CpG when mixed with only excipient ELP (“All excipient”), freely migrates through the agarose gel (**Figure** **1**A), indicating that the excipient ELP—as expected—has no electrostatic interactions with the CpG. The “All Lys_12_” control exhibited near‐complete retardation of CpG in the gel, while the 100, 80, 60, and 40 lanes show, by comparison, a continuously decreasing proportion of CpG remaining in the loading well. Quantitative analysis of the bound‐ versus unbound‐CpG fraction for each sample show clearly increasing proportions of free, unbound CpG as the amount of ELP‐Lys_12_ is decreased (Figure 1B). This data demonstrates that our strategy of replacing ELP‐Lys_12_ with excipient ELP affects the binding of CpG to ELP‐Lys_12_.

**Table 1:** Composition of ELP‐Lys_12_, excipient ELP, and CpG depots. 100, 80, 60, and 40 ELP‐CpG formulations comprise a total ELP concentration of 1044 µм (52.2 nmols) and a CpG concentration of 313 µм (15.66 nmols). N:P ratio refers to the ratio of positively charged amine groups in the ELP‐Lys_12_ to the negatively charged phosphate backbone of the CpG DNA.

Formulation	nmols ELP‐Lys_12_	nmols Excipient ELP	nmols CpG	N:P Ratio
**100**	26.1	26.1	15.66	1
**80**	20.88	31.32		0.8
**60**	15.66	36.54		0.6
**40**	10.44	41.76		0.4
**ELP‐Lys_12_**	26.1	0		1
**Excipient ELP**	0	52.2		0
**CpG only**	0	0		N/A

We then confirmed the bioactivity of ELP‐CpG in vitro using a Griess nitrite quantification assay. RAW264.7 is a murine‐derived macrophage cell line that is highly sensitive to pattern‐associated molecular patterns (PAMPs) such as those found in CpG.^[^ ^17^ ^]^ When CpG is administered to cultured RAW264.7 cells, they produce nitric oxide (NO), the level of which is proportional to the degree of CpG stimulation.^[^ ^17^, ^18^ ^]^ In RAW264.7 cells stimulated with soluble CpG or ELP‐bound CpG, we found a significantly elevated (3.5‐fold increase) level of nitrite production compared to a PBS control (Figure 1C). Furthermore, CpG bound to ELP‐Lys_12_ exhibited a significantly greater activity (11.5% increase) than soluble CpG, an expected finding, given our previous work demonstrating improved cellular internalization of ELP‐CpG nanocomplexes over that of soluble CpG.^[^ ^14^ ^]^

**Figure 2:**
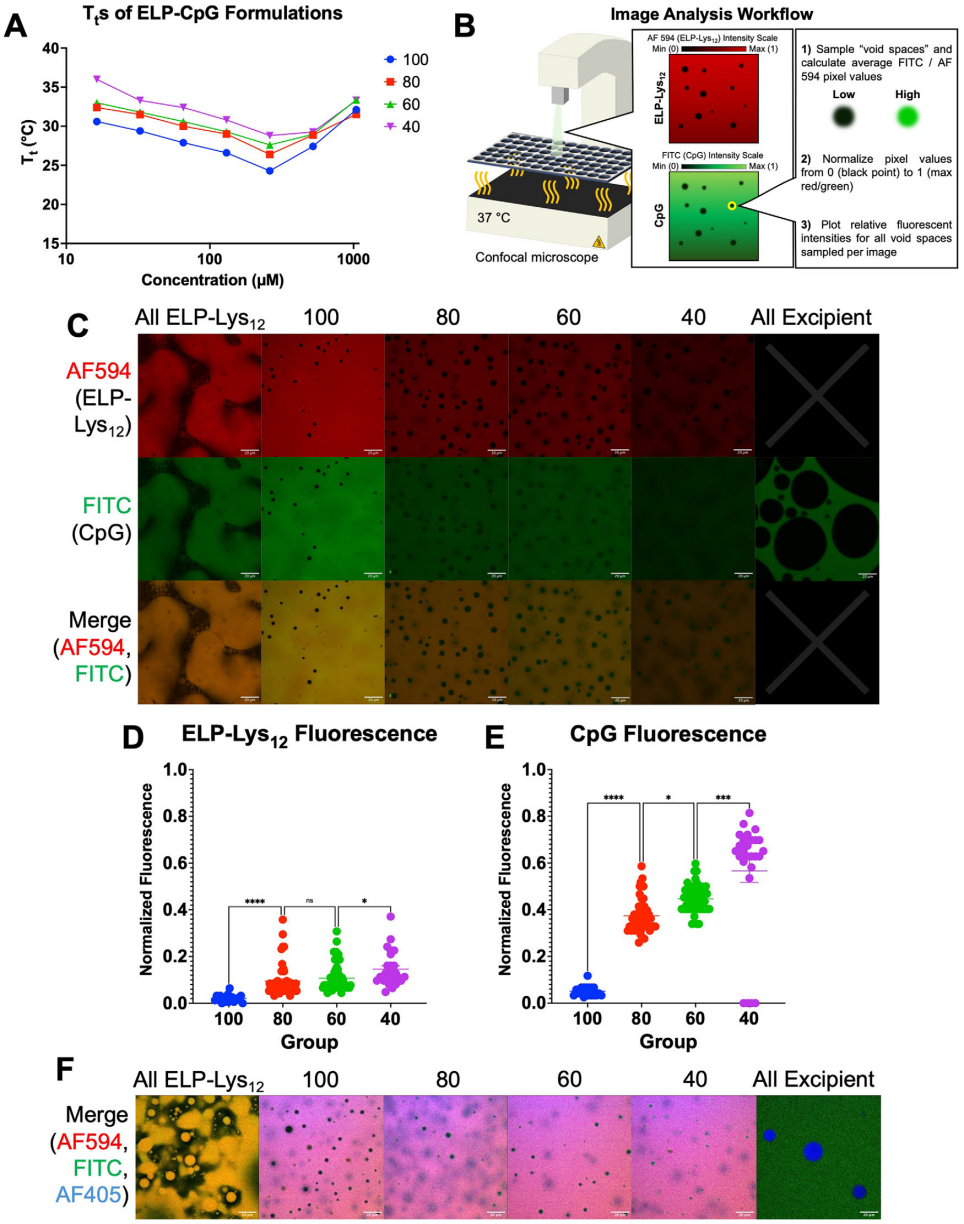
A) Transition temperature (T_t_) of ELP‐CpG formulations (ELP‐Lys_12_ and excipient ELP) as a function of the total ELP concentration in the formulation. B–E): Mixtures of ELP‐Lys_12_ with excipient ELP and CpG at 37 °C phase‐separate into ELP‐poor “void spaces” throughout depots that are rich in soluble CpG. (B) Workflow for image analysis of “void spaces” within ELP‐CpG depots. (C) Confocal microscopy images of seven formulations (All ELP‐Lys_12_, 100, 80, 60, 40, and All Excipient) consisting of AF594‐ELP‐Lys_12_ (top row), FITC‐CpG (middle row), and a merge of the two channels (bottom row). Scale bar: 20 µm. (D) Normalized fluorescence intensities for AF594‐ELP‐Lys_12_ and (E) FITC‐CpG in the voids in panel B. ****p < 0.0001, *p < 0.05 (ANOVA, Tukey). F) Confocal microscopy composite images of seven formulations comprising AF594‐labeled ELP‐Lys_12_, FITC‐labeled CpG, and AF405‐excipient ELP (full set of images for individual channels in Figure S13, Supporting Information).

We next characterized the thermally responsive LCST phase behavior of ELP‐CpG (Figure S7, Supporting Information). Samples were prepared at seven concentrations at the same ratios as described above (calculated as ELP‐Lys_12_ plus excipient ELP concentration): 1044, 522, 261, 130.5, 65.25, 32.625, and 16.3125 µм. Upon heating from 15 °C to 40 °C, and subsequently cooling back to 15 °C, we observed that all samples showed LCST phase behavior with a T_t_ between 25 °C and 37 °C, T_t_s that will allow for depot formation upon injection in vivo (**Figure** **2**A). Furthermore, the phase transition of all samples was fully reversible upon cooling. Importantly, we observed that as the fraction of ELP‐Lys_12_ in each formulation decreased, the overall T_t_ increased, which is a predictor of faster depot dissolution kinetics in vivo.^[^ ^19^ ^]^

We used fluorescence microscopy to visualize the microdroplets formed by ELP‐CpG. Samples (all ELP‐Lys_12_, 100, 80, 60, 40, and all excipient) were prepared on ice at a total ELP concentration of 1044 µм and a CpG concentration of 313.2 µм. The ELP‐Lys_12_ and CpG were doped with 2% and 1% (v/v) Alexa Fluor 594 labeled ELP‐Lys_12_ (AF594‐ELP‐Lys_12_) and fluorescein isothiocyanate labeled CpG (FITC‐CpG), respectively. Upon heating to 37 °C, we observed immediate liquid‐liquid phase separation and subsequent droplet formation in all samples (Figure S9, Supporting Information). We observed small droplet nuclei fusing into larger droplets until settling into large coacervate puddles over several minutes. We also observed colocalization between the AF594‐ELP‐Lys_12_ and FITC‐CpG, indicating complexation between the ELP and CpG across the droplets. Additionally, we observed significantly more background FITC signal as the proportion of ELP‐Lys_12_ decreased in each formulation, indicating that there is more unbound (soluble) CpG present in these samples beyond the ELP‐rich droplets. We used fluorescence recovery after photobleaching (FRAP) to see whether the larger, settled protein coacervate of ELP‐CpG was also liquid‐like. Upon photobleaching of the protein “puddle,” we observed minimal FRAP recovery in all ELP‐CpG samples, indicating that once the ELP‐CpG droplets fuse into a single large ELP‐rich depot, they are much more solid‐like than liquid‐like (Figure S10, Supporting Information).

We next investigated the microscale morphology of each ELP‐CpG formulation. To do so, we mixed identical formulations as above, using AF594‐ELP‐Lys_12_ and FITC‐CpG, plated them onto a 384‐well plate, and heated the mixtures to 37 °C. We then imaged the resulting coacervates using confocal laser‐scanning microscopy. The images demonstrate that ELP‐CpG mixtures undergo microphase separation into holes—“void spaces”—to varying degrees depending on composition. The workflow for image analysis and quantification of the composition of the void spaces within ELP‐CpG depots is shown in Figure 2B. A sample of only ELP‐Lys_12_ (no excipient) mixed with CpG exhibited complete colocalization of ELP‐Lys_12_ and CpG within the arrested droplets (Figure 2C, bottom left). When excipient ELP was added, however, unbound soluble CpG phase separated into numerous, spherical void spaces distributed throughout the 100, 80, 60, and 40 formulations (Figure 2C). These regions, much darker than the surrounding ELP‐Lys_12_‐rich area, appear nearly devoid of ELP‐Lys_12_, but contain visually discernable amounts of soluble CpG (Figure 2C). As the proportion of excipient ELP in the mixture increased, the amount of soluble CpG in these spherical regions also increased. We quantitatively confirmed this observation by calculating the fluorescence signal for AF594 and FITC in the spheres within a single imaging region and normalizing it to a scale set between the lowest and highest fluorescent intensity values for each image (Figure 2D,E). We then analyzed the morphology of the void spaces using ImageJ and found that both the diameter and area of the void spaces in the 80, 60, and 40 formulations is significantly higher than the 100 formulation (Figure S11, Supporting Information). In addition to populations of voids that have similar diameter and area to those in the 100 group, we noticed that the other ELP‐CpG formulations exhibited additional populations of much larger voids.

These results suggest there is phase separation of soluble CpG in the presence of the excipient ELP. Within this ternary system, there are multiple phases in which the phase‐separated CpG can exist. First, there is the bound‐CpG that is colocalized with ELP‐Lys_12_ (yellow regions in Figure 2C, merge channel). We hypothesize that in these regions, colocalization of CpG and ELP is driven by the electrostatic interaction between the phosphate backbone of the DNA and the positively charged Lys residues on the ELP. When only ELP‐Lys_12_ and CpG are mixed, this is the case for nearly all the ELP and CpG within the solution (Figure 2C, “All ELP‐Lys_12_,” Merge channel). We also hypothesize that in the absence of excipient, ELP‐Lys_12_ and CpG alone undergo more rapid droplet fusion into the coacervate phase, precluding the ability for the “void spaces” to form in this sample. Upon the addition of excipient ELP, however, void spaces arise due to microphase separation of the aqueous, CpG‐rich phase from the surrounding ELP phase. This is suggested by Figure 2C (“All Excipient” sample, FITC channel), which shows a pool of FITC‐CpG that is interrupted by large droplets devoid of CpG. If this is the case, it confirms that the excipient ELP drives the unbound CpG (of which there is increasingly more as the proportion of ELP‐Lys_12_ decreases) to phase‐separate into higher‐concentration regions of soluble DNA.

To confirm the hypothesis that the “void spaces” are reservoirs of soluble CpG and are lacking both ELP‐Lys_12_ as well as excipient ELP, we repeated the above experiment, but in addition to the two previously labeled components, we labeled the excipient ELP with Alexa Fluor 405 (AF405) at a 1% (v/v) ratio. Upon heating to 37 °C, we observed identical void spaces in the excipient ELP channel as was present in the ELP‐Lys_12_ channel (Figure 2F). Further, the excipient ELP phase‐separates into droplets that selectively exclude CpG (Figure 2F, “All Excipient” sample). This data unequivocally confirms that ELP‐CpG nanocomplexes—in the presence of excipient ELP—phase separate into: 1) ELP‐rich regions with bound CpG; and 2) spherical CpG‐rich and ELP‐poor regions that are reservoirs for soluble CpG to quickly diffuse from the coacervate. The mechanism of this behavior—of which similar instances have been observed in other biomolecular condensates^[^ ^20^ ^]^—could be a kinetic process in which, as the ELP coacervate forms under heat, the solidification of the biopolymer slowly expels water (and CpG) from within the ELP matrix, which accumulates within the void spaces.

We next sought to investigate the nanoscale structure of this ternary system—ELP‐Lys_12_, excipient ELP, and CpG—using small‐angle X‐ray scattering (SAXS). We prepared samples of the ELP‐CpG 100 formulation (as described in Table 1) as well as the ELP‐Lys_12_, excipient ELP, and CpG components, and a separate sample of ELP‐Lys_12_ with CpG. We arrived at several conclusions after analysis of the SAXS data. First, at room temperature, excipient ELP and ELP‐Lys_12_ each exhibit completely unfolded, random chain structures with an R_g_ of 5 nm (Figure S12A,B, Supporting Information), while CpG exhibits signs of folded structures indicative of dsDNA (Figure S12C, Supporting Information). At room temperature, mixtures of ELP‐Lys_12_ and CpG, while exhibiting confirmational changes of both components indicative of electrostatic complexation, do not demonstrate evidence of higher order self‐assembly, with an R_g_ of 5 nm (Figure S12D, Supporting Information). When all three components are mixed, we observed a concentration‐dependent correlation peak at q = 0.034 Å^−1^, corresponding to 18.5 nm in real space (Figure S12E, Supporting Information). A correlation peak appears when there is periodic distance or ordered structure within the sample.^[^ ^21^ ^]^ Coupled with our previous observation that the ternary system drives CpG to microphase separate—leading to local high‐density regions of DNA—we hypothesize that the distance of 18.5 nm is between the charged CpG domains. When the sample of ELP‐Lys_12_, excipient ELP, and CpG is heated to 37 °C, the correlation peak shifts to q = 0.028 Å‐1, corresponding to 22.4 nm in real space (Figure S12F, Supporting Information). We hypothesize this is indicative of the ELP coacervate collapsing as it phase‐separates from the surrounding water, which then increases spacing of the DNA chains within the microdroplets.

### 2.3 In Vivo Evaluation of ELP‐CpG in the 4T1 Breast Cancer Model

We hypothesized that tuning the fraction of CpG bound to ELP‐Lys_12_ versus free CpG would modulate the release kinetics of CpG from the depot, as free CpG from the void spaces would show burst release upon *i.t*. injection, whereas the ELP‐bound CpG would be released at a slower rate from the depot. To measure the release of CpG from an *i.t*. depot, we chose the murine 4T1 mammary carcinoma model, as we could establish orthotopic tumors that would be easily imaged using whole‐body in vivo fluorescence imaging, while being also therapeutically relevant, as it is a syngeneic model. Female BALB/c mice (n = 5) were inoculated orthotopically with 4T1 tumors and—once tumors grew to 100 mm^3^—treated *i.t*. with either 100, 80, 60, or 40 formulations of ELP‐CpG, or with a soluble CpG control. All treatment groups contained 100 µg of CpG, 2% (v/v) of which was fluorescently labeled at the 3′‐terminus with Alexa Fluor 647 (AF647) dye. After injection, we collected whole‐body fluorescence images at t = 0, 1, 3, 6, 12, 24, 48, 72, and 96 h, and subsequently every 48 h, up to t = 624 h. The average radiant efficiency within the depot was then calculated and compared as a fraction of the radiant efficiency 1 h after injection (to account for depot‐formation and leakage from the tumor after needle withdrawal).

**Figure 3:**
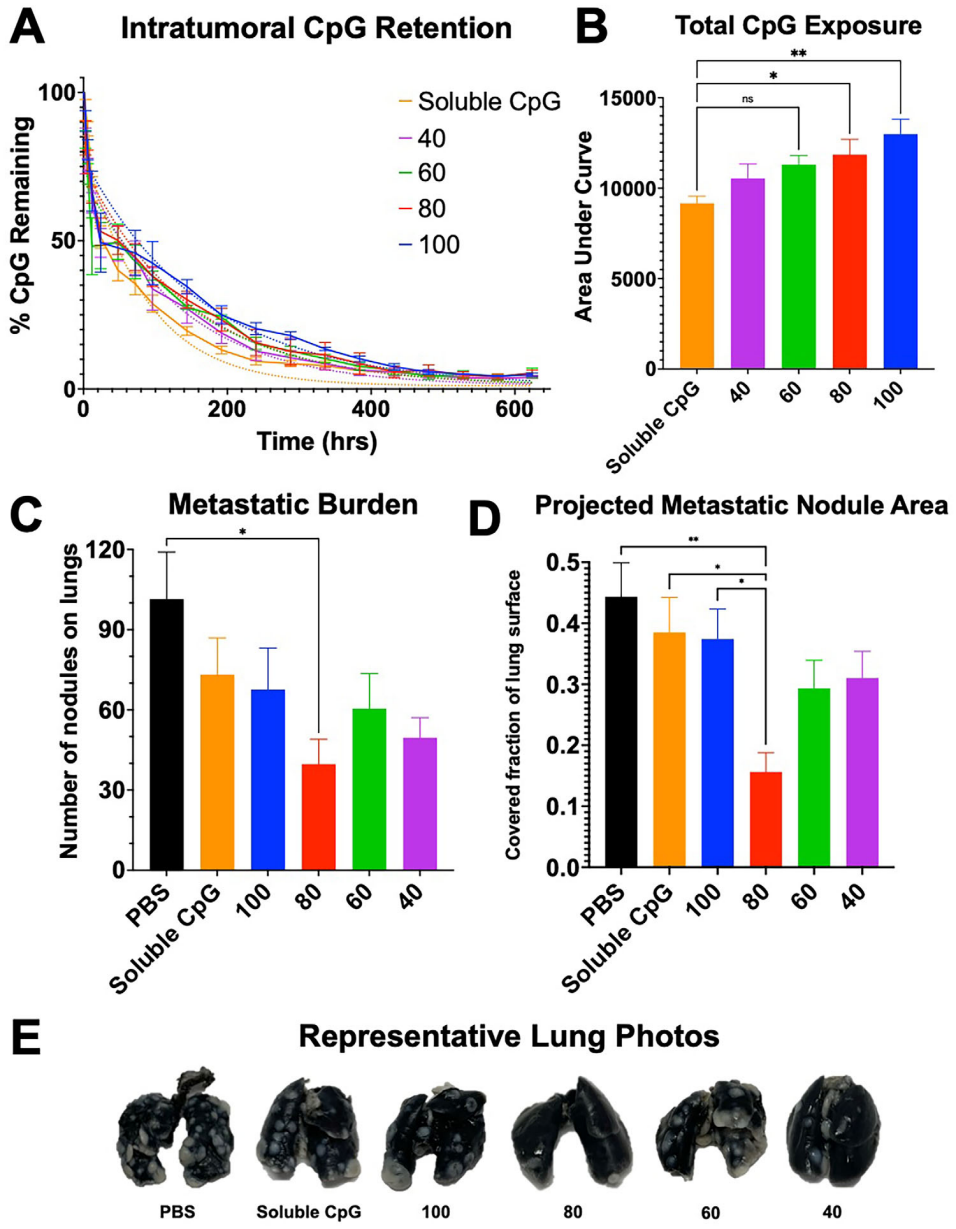
ELP‐CpG nanocomplex prolongs retention of CpG within tumors and reduces proliferation of lung metastases in mice with metastatic mammary carcinoma. A) Plot of *i.t*. CpG retention as a fraction of the average radiant efficiency of the injected CpG depot in each mouse at t = 1 h after injection. Dotted line: curve fit of biphasic release model for each CpG formulation as described in Equation (1). B) Total *i.t*. CpG exposure for each treatment group as measured via area‐under‐curve (AUC) analysis from Figure 3A. Error bars represent ± one standard error of the mean (SEM). C) Mean number of lung metastatic nodules among mice (n = 7‐10) treated with PBS control or CpG (soluble or ELP‐bound). Error bars represent ± one standard error from the mean (SEM). D) Fraction of projected tumor area on lung surface as determined using image analysis of stained lungs. E) Representative photos of lung pairs from each group (metastatic nodules appear lighter in color compared to surrounding dark‐stained lung tissue). *p < 0.05, **p < 0.01 (ANOVA, Tukey).

The imaging results show that ELP‐bound CpG significantly enhances *i.t*. CpG retention compared to soluble CpG alone (**Figure** **3**A). As expected, soluble CpG was cleared faster than any ELP‐bound formulation, with the 40, 60, 80, and 100 ELP‐CpG groups exhibiting progressively greater CpG retention over the following weeks. Area under curve (AUC) analysis demonstrates that there is no significant difference in CpG retention between the soluble CpG and the two weakest‐binding ELP groups (40 and 60), but there is a significant difference between those and the 80 and 100 groups (Figure 3B). As seen in Figure 3A, all ELP formulations exhibit similar dissolution of CpG from within the tumor over the first 24–48 h, decreasing to ≈50% of the original CpG intensity. After this point, however, the ELP groups retain progressively more CpG in accordance with the expected binding strengths of each formulation.

To better analyze the pharmacokinetics of ELP‐CpG formulations compared to soluble CpG, we fit the *i.t*. retention data to a biphasic release model^[^ ^22^ ^]^ (assuming release of CpG from a tumor occurs in both a burst and sustained‐release phase) using Equation (1) below. The empirical equation is based on similar equations modeling a combination of Fickian and non‐Fickian diffusion,^[^ ^22^ ^]^ which we hypothesize occurs in the case of ELP‐bound CpG within a tumor. The fraction of CpG remaining in the tumor at time *t* is denoted by *M_t_*.

(1) $$M_{t}=A\left(1-e^{-k_{1}t}\right)+B\left(1-e^{-k_{2}t}\right)$$

The parameters fit within Equation (1) include: A (fraction of CpG released quickly via burst), B (fraction of CpG released slowly through the depot/tumor matrix), and k_1_ and k_2_ (time‐rate constants for each release phase). The parameters were constrained to improve the goodness‐of‐fit (*R* ^2^ > 0.85) of the resulting model for each formulation. Constraints used for the parameters were:

(2) $$A<0\to 0<k_{1}<10\to 0<k_{2}<1$$

The release behavior we observed generally follows the biphasic release model in Equation (1), apart from a period roughly from 24 to 72 h following injection. In this period, the modeled (expected) fraction of CpG remaining in the depot is higher than the observed fraction. We hypothesize this phenomenon that is due to the initial depot formation occurring rapidly following injection, where the ELP coacervate forms in a milieu of interstitial fluids entering/exiting the tumor that remove some fraction of the CpG immediately from within the tumor before the ELP depot has heated to body temperature and fully stabilized.

After confirming that we can precisely tune *i.t*. CpG retention, we tested the ELP‐CpG formulations as a monotherapy to see whether a single, “optimal” formulation would yield better efficacy. We evaluated therapeutic efficacy using the same 4T1 model, which would allow us to observe the effects of tuning CpG release on metastatic burden after treatment. Female BALB/c mice (n = 8‐10) were inoculated orthotopically with 4T1 tumors and—once tumors grew to 100 mm^3^—treated *i.t*. with either the 100, 80, 60, or 40 formulations described above, or with PBS/soluble CpG controls. All treatment groups contained 100 µg of CpG. We observed little change in the growth rate of the primary tumor for the CpG‐treated groups compared to the control (Figure S15, Supporting Information). Median survival (Figure S16, Supporting Information) increased slightly to a maximum of 27 days in the ELP‐CpG 80 group (compared to 25.5 days in the control).

In parallel, we performed the same experiment but took a “snapshot” of the *i.t*. and systemic immune landscape, as well as the spread of lung metastases. In this experiment, mice were sacrificed when one third of the PBS control group reached humane endpoint criteria. Upon sacrifice, lungs were excised and stained with India ink. They were then washed and fixed with Fekete's solution, upon which visible lung metastases were counted (Figure 3C,D). We observed a decrease in the number of lung metastases in the CpG‐treated groups, particularly in the 80 group (Figure 3C). Using image analysis to compare the area of visible metastatic nodules compared to the area of the whole lung pair, the projected metastatic lung area was significantly lower for the 80 group compared to the PBS, soluble CpG, and 100 groups (Figure 3D). Additionally, the number of individual metastatic nodules in the 80 group was significantly less than that of the PBS control (40 vs 93 nodules per lung pair, respectively).

In addition to analyzing lung metastatic burden, we harvested the tumors and spleens, processed them into a single cell suspension, stained them with antibodies for CD45 (lymphocytes), CD11c (dendritic cells), CD86 (costimulatory molecule), and MHC Class II, and used flow cytometry to quantify differences in expression of these markers. In the tumor, we observed no changes in CD45^+^ cell count, frequency, or expression among the groups (Figure S17, Supporting Information). There was no apparent difference in CD11c^+^ cell count or frequency among the groups, besides a decrease in CD11c expression among the soluble CpG and 40 groups (Figure S18, Supporting Information). There was a trend in which the MHC class II^+^ cell frequency was elevated to the greatest degree in the 80 group compared to the PBS, ELP‐CpG 40, and soluble CpG groups (Figure S19, Supporting Information). Mean MHC class II^+^ cell frequency was also higher among all CpG‐treated groups than in the PBS control. The spleens were notably enlarged because of the splenomegaly associated with 4T1.^[^ ^23^ ^]^ There was a slight increase in CD11c expression and CD86^+^ cell frequency in the 80 group (Figure S21, Supporting Information), and there was slightly higher CD11c expression, CD86^+^ cell frequency, and MHC Class II^+^ frequency in splenocytes from the 80 group (Figure S21, Supporting Information). These data can only be considered trends at best, as none of the changes were statistically significant.

### 2.4 Early After Treatment, ELP‐CpG Suppresses Lymphatic Invasion of 4T1 and Enhances i.t. Immune Cell Engagement

Based on the findings from our previous flow cytometry experiment, where all mice were sacrificed once a fraction of the PBS control mice reached their humane endpoint 18 days post‐treatment, we expect that ELP‐CpG suppresses proliferation of 4T1 lung metastases by virtue of expanding DC signaling, antigen‐presentation on MHC class II, and improving cross‐presentation of tumor‐associated antigens to cytotoxic CD8^+^ T cells within the tumor. To further investigate this hypothesis, we performed an additional study in which mice (n = 10 per group) were treated as before with either PBS (control), soluble CpG, or the optimized ELP‐CpG (80) formulation sacrificed three days post‐treatment, and the tumors were excised, homogenized, and analyzed with an expanded flow cytometry panel that comprises: CD45, CD11c, CD86, and MHC class II as before, but additionally CD8 (cytotoxic T cells) and F4/80 (a macrophage marker).

**Figure 4:**
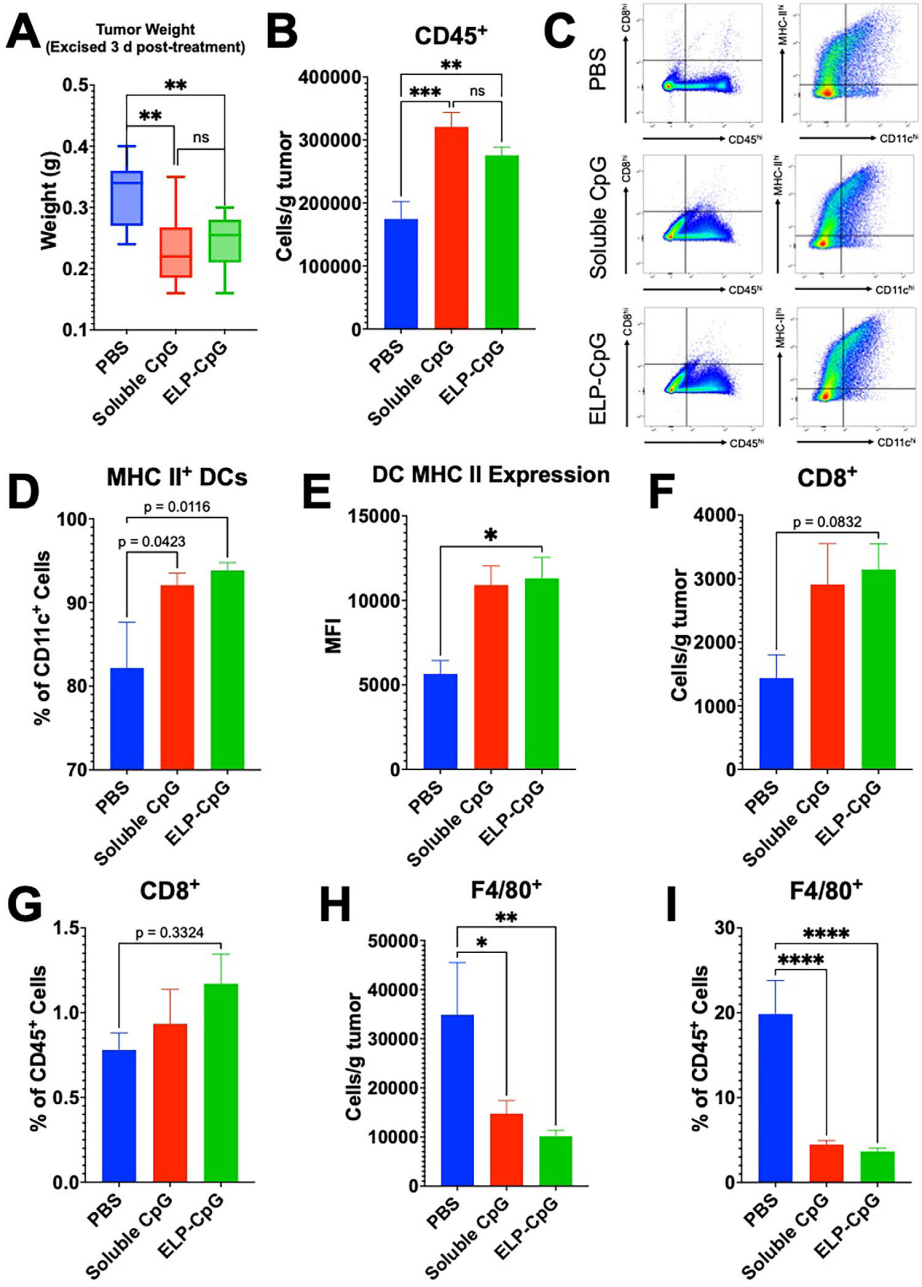
ELP‐CpG alters the immune cell makeup and activation states within the *i.t*. environment. A) Weight of 4T1 tumors excised from mice (n = 9‐10) 3 d following treatment with either PBS (blue), soluble CpG (red), or ELP‐CpG (green). Tumor samples were then digested, homogenized into a single cell suspension, and analyzed with flow cytometry for *i.t*. immune cells (PBS n = 5, soluble CpG n = 7, ELP‐CpG n = 9). B) Weight‐normalized number of CD45^+^ cells. C) Representative dot plots for CD45^+^CD8^+^ T cell (left) and CD11c^+^MHC‐II^+^ activated dendritic cell populations. D) MHC class II‐expressing DCs as a fraction of total DCs and, E) expression levels of MHC class II on DCs. F) Weight‐normalized number of CD8^+^ T cells and, G) CD8^+^ T cells as a fraction of all lymphocytes. H) Weight‐normalized number of F4/80^+^ cells (macrophages) and, I) F4/80^+^ macrophages as a fraction of all lymphocytes. Error bars: one standard error from the mean (SEM). ns: not significant, *p < 0.05, **p < 0.01, ***p < 0.001, and ****p < 0.0001 (ANOVA, Tukey).

Immediately following sacrifice, we weighed the tumor samples and found that in mice treated with soluble or ELP‐CpG, tumor weights were significantly lower than the PBS control (**Figure** **4**A). This indicates that for at least a brief period following treatment, CpG and ELP‐CpG can suppress tumor growth compared to a PBS control. We discovered during excision that in several mice, there was noticeable invasion of the tumor into the nearby draining inguinal lymph node (dLN), which impeded separation of the two structures. Invasion of tumor cells into the nearby dLN is a hallmark of 4T1, which occurs shortly following tumor implantation and continues remodeling the lymphatic vasculature and structure throughout disease.^[^ ^24^ ^]^ We observed that, of the excised tumor samples from each group, half of all PBS‐treated mice (5/10) exhibited infiltration of tumor into the dLN while only 3/10 soluble CpG‐treated mice and 1/10 ELP‐CpG‐treated mice exhibited this infiltration. With flow cytometry, we also observed a large contaminating lymphocyte population in these samples and on this basis, we excluded these samples from subsequent analysis. These findings suggest that *i.t*. CpG therapy (and to a greater extent, ELP‐CpG) suppresses lymphatic invasion and remodeling (at least at the timepoint analyzed in this experiment), which we hypothesize slows the pathway for cancer cells to metastasize to the lungs. We analyzed tumor homogenates with ELISAs for CpG and interferon‐γ (Figure S22, Supporting Information), and found that while *i.t*. IFNγ levels were higher in soluble CpG than ELP‐CpG mice, this could be a consequence of burst release of soluble CpG, as levels of ELP‐bound CpG were still higher at that time than in the soluble CpG group.

We observed a significantly elevated fraction of CD45^+^ cells in the soluble CpG and ELP‐CpG groups compared to PBS (Figure 4B). The weight‐normalized fraction of CD45^+^ cells for soluble CpG was not significantly higher than ELP‐CpG, which was surprising as we had hypothesized that it would be higher at the three‐day timepoint due to greater burst release of soluble CpG within the tumor at that point, boosting the immediate response to soluble CpG to a higher degree than ELP‐CpG. Of tumor‐infiltrating lymphocytes, the fraction of both CD11c^+^ and CD86^+^ DCs was elevated in soluble CpG and ELP‐CpG groups (Figure 4C), and these were consistent trends throughout the data set (Figures S23 and S24, Supporting Information). MHC class II^+^ DCs were significantly expanded (as a fraction of DCs) in soluble CpG and ELP‐CpG groups (Figure 4D). We also found that expression of MHC class II on DCs was significantly higher in both CpG‐treated groups and was elevated slightly higher in the ELP‐CpG group (Figure 4E).

Tumor‐infiltrating CD8^+^ T cells were also notably expanded in the CpG‐treated groups (Figure 4C) compared to PBS (again, with trends mirroring other findings showing greater engagement in the ELP‐CpG group, Figure 4F,G). Finally, we found that the weight‐normalized number of F4/80^+^ tumor‐associated macrophages was substantially lowered in the soluble CpG group, and even more so in the ELP‐CpG group (Figure 4H). The fraction of CD45^+^ cells also expressing F4/80 (Figure 4I) exhibited an even greater difference in CpG treated mice (compared to PBS control), suggesting that CpG (and to a greater degree, ELP‐CpG) induces a significant reduction in resident tumor‐associated macrophages that, we hypothesize, are anti‐inflammatory and/or tumorigenic, though confirming this requires additional investigation.

## 3 Discussion

Immunotherapy holds promise as the future of effective metastatic cancer treatment. As conventional therapeutics are minimally effective against metastatic, solid tumors,^[^ ^25^ ^]^ new drug delivery approaches continue to be investigated.^[^ ^26^ ^]^ Immunogenic agents such as CpG are effective through their uptake by dendritic cells, within which they stimulate the production of type‐I interferons.^[^ ^9^, ^10^, ^27^ ^]^ Additionally, TLR9‐activated DCs undergo maturation and increased expression of MHC class II molecules on their surface,^[^ ^28^ ^]^ bridging the gap between innate and adaptive immunity.^[^ ^29^ ^]^ Generally, cancer neoantigens are poorly immunogenic on their own; furthermore, immunologically “cold” tumors contain a myriad of immunosuppressive cytokines and cells that present a high barrier against sustained antigen‐presenting cell signaling and activation.^[^ ^30^ ^]^

To enhance activation of DCs within the intratumoral space, we previously developed ELP‐CpG to greatly extend CpG retention within the local site.^[^ ^14^ ^]^ This achieved the desired effect of enhancing the systemic immune response against 4T1 tumors, as mice treated with ELP‐CpG exhibited fewer metastases compared to those treated with soluble CpG.^[^ ^14^ ^]^ ELP‐CpG also has the advantage of enhancing CpG internalization within APCs. Building on this work, herein we hypothesized that while increasing CpG retention in a tumor would enhance its exposure to intratumoral APCs, DC‐mediated uptake, and systemic immune engagement, there is a point at which slowing CpG release might diminish the immune response. Previous studies have shown that the timing of adjuvant CpG in cancer therapy affects the overall efficacy.^[^ ^7d^ ^]^ Motivated by these observations, we sought to tune the release of CpG from our ELP depot and evaluate the effects on both retention and therapeutic response.

ELPs enable highly rational tuning of drug retention and release from a depot. Our group has shown that sequence‐level changes to the polypeptide backbone (primarily length and guest amino acid residue composition) causes precise, predictable changes to the transition temperature of the ELP.^[^ ^31^ ^]^ However, the effects on mixing different ELPs to achieve a similar effect have not been previously studied. We chose ELP‐Lys_12_ as one of the two ELPs in the mixture and the same ELP without the Lys_12_ tag as the excipient and varied the ratio of the two but with the total ELP concentration held constant across all mixtures. We extensively characterized the effects of varying the molar ratio of excipient ELP to ELP‐Lys_12_. As expected, reducing the amount of ELP‐Lys_12_ and replacing it with an equivalent amount of excipient ELP reduced the amount of charge‐neutralized, ELP‐bound CpG in the ternary system of ELP‐Lys_12_, ELP and CpG. Excipient ELP exhibits a higher T_t_ than ELP‐bound CpG, meaning that as the proportion of excipient ELP in each formulation increases (100, 80, 60, and finally to 40), the T_t_ of the system also increased. Interestingly, the T_t_ was not a function of concentration, with the phase‐diagram of the ELP‐CpG mixtures exhibiting behavior like single‐ELP depots at concentrations ranging from 16.3125 µм to 1044 µм.

Visualization of the microstructure of this ternary system consisting of ELP‐Lys_12_, CpG, and excipient ELP by laser scanning confocal fluorescence microscopy showed that at 37 °C, the ELP‐Lys_12_ on its own forms distinct ELP‐ and CpG‐rich droplets. Conversely, formulations containing excipient ELP underwent droplet‐fusion into large amalgamations with clearly defined, near‐uniform “holes” in the ELP puddle. We observed these void spaces preferentially exclude CpG as the proportion of ELP‐Lys_12_ in the formulation increased, a finding that we quantitatively confirmed using image analysis. We hypothesized that as the proportion of unbound CpG increased and the amount of ELP‐Lys_12_ decreased, the void spaces would be analogous to pores in a sponge, where more soluble CpG present in the holes could be quickly released from a depot. We repeated this experiment with fluorescently labeled excipient to confirm that the holes were not only preferentially excluding ELP‐Lys_12_, but also excipient ELP, meaning that the CpG‐rich pockets in each coacervate function as CpG‐rich, ELP‐poor reservoirs for soluble CpG to be released.

We investigated the internal nanoscale structure of the depot by SAXS, which revealed that below the T_t_, a ternary system of ELP‐CpG exhibits a nearest particle distance of 18.5 nm that—we hypothesize—arises from microphase separation between ELP chains and unbound CpG. When heated to body temperature, the collapse of the ELP allows for larger DNA droplets and hence the nearest particle distance increases to 22.4 nm. The microphase separation observed here warrants further investigation to understand the mechanism that drives the formation of these nanostructured domains, and thereby learn how to tune this behavior to further optimize this delivery system and potentially expand it to other therapeutic modalities.

Based on our in vitro characterization, we expected that ELP‐CpG would release CpG from an intratumoral depot more quickly as the ratio of excipient ELP to ELP‐Lys_12_ increases. We tested this using IVIS fluorescent imaging, where we showed that all ELP‐bound formulations increase the cumulative *i.t*. CpG exposure over soluble drug. ELP‐bound formulations retained more CpG particularly between 2–12 days after treatment. As expected, the 100 group retains the highest amount of CpG (as measured by AUC), followed by the 80, 60, 40, and soluble CpG groups.

Having confirmed that changing the ELP formulation affects the *i.t*. retention of CpG in a predictable, tunable way, we used a therapeutic model to see if optimized ELP‐CpG could reduce lung metastatic burden in 4T1 tumor‐bearing mice. 4T1 is a challenging tumor model for immunotherapies due to its rapid metastatic spread and poor expression of immunogenic neoantigens.^[^ ^32^ ^]^ For these reasons, a tunable, sustained‐release formulation of CpG is ideal for increasing immune cell engagement with otherwise poorly immunogenic tumor antigens and maximizing the release of CpG within the optimal therapeutic window for the fast‐growing tumors. We found that different ELP‐CpG formulations affected therapeutic efficacy by reducing metastatic spread in the lungs of tumor‐bearing mice. While the 100 and soluble CpG groups do not exhibit notable reduction of metastatic spread on their own, the 80, 60, and 40 groups more notably reduce the number of lung metastatic nodules, with the 80 group doing so most significantly. Although the ELP‐CpG monotherapy reduced metastatic disease, there was no significant extension of survival in these studies. This was due to continued and, ultimately, fatal growth of the primary tumors in all mice, which grew to humane endpoint criteria rapidly. As 4T1 tumors are extremely fast growing, it is likely that the necrotic core of the tumor (where the CpG was injected) exhibited a different immune cell landscape and tumor microenvironment than the fast‐growing periphery of the tumor. We also hypothesize that, due to the highly immunosuppressive nature of distant metastases in models such as 4T1,^[^ ^33^ ^]^ distant sites of metastasis in the brain, liver, and bones^[^ ^34^ ^]^ (among other sites) could be drivers for death even in mice which do not exhibit evidence of significant lung disease. There remains strong rationale for combining intratumoral ELP‐CpG with a means of local tumor control, such as ELP brachytherapy,^[^ ^14^, ^35^ ^]^ to stymie tumor growth while antitumor immunity develops systemically thanks to the adjuvant CpG. We anticipate that an optimized formulation of ELP‐CpG, as demonstrated here, could synergize with local brachytherapy to improve efficacy beyond what we have previously observed in studies combining an unoptimized ELP‐CpG formulation with brachytherapy.^[^ ^14^ ^]^

In this study, we further analyzed the effects on antitumor immunity beyond lung metastatic spread alone. Harvesting cells from both the tumors and spleens, we observed trends in immune cell recruitment that demonstrate the effects of optimizing local CpG release on systemic immunity. CD11c expression was lower in the soluble CpG and 40 groups. CD11c down‐regulation may occur upon activation of TLR9,^[^ ^36^ ^]^ so it is possible that the *i.t*. APCs in these groups were still exhibiting the effects of substantial and rapid CpG exposure after the injection. An alternative explanation is that additional CD11c^+^ cells were recruited to the tumor in the 100, 80, and 60 groups as the extended, more balanced release of CpG occurred, leading to a net increase in CD11c expression. We also observed that the 80 group exhibited the greatest increase in MHC Class II^+^ cell frequency. The expression of MHC Class II on the MHC‐II^+^ cells was also elevated in the 80 group. The effects on the immune landscape among splenocytes were similar as to what we observed intratumorally: increased CD11c expression, CD86^+^ cell frequency, and MHC‐II^+^ cell frequency are all indicators of increased DC and broader APC activation and presentation of tumor‐associated antigens.

We confirmed these findings are a sustained result of early‐stage (3 d following treatment) immune cell activation and remodeling of the tumor microenvironment that occurs due to CpG treatment. Specifically, we found that at this stage, CpG (soluble or ELP‐bound) had a clear effect on tumor weight, but given our observations from the immunoprofiling carried out later at 18 d post‐treatment, these effects are not a durable suppressor of tumor growth over multiple weeks. We did find that ELP‐CpG treated mice had, overall, fewer instances of tumor invasion into the dLN (1/10 mice) than either soluble CpG (3/10 mice) or PBS (5/10 mice). We discovered that despite retaining a greater fraction of CpG within the tumor at 3 d as compared to the soluble CpG treatment, ELP‐CpG still induces greater activated DC levels and greater expression of MHC class II within the tumor DC compartment. We also observed that greater numbers of cytotoxic CD8^+^ T cells are present in the tumors of ELP‐CpG treated mice, cells that are important for suppression of metastatic disease. Finally, we found striking differences in the number of tumor‐associated macrophages in mice treated with PBS, soluble CpG, or ELP‐CpG. We hypothesize that these macrophages are a driver of immunosuppression, particularly in the PBS group, and thus may promote tumorigenesis and metastatic spread. Immunoprofiling at three days post‐treatment supports the hypothesis that, while soluble CpG supports a greater initial burst release of CpG—which coincides with higher proinflammatory IFNγ signaling and immediate DC activation—ELP‐CpG ultimately imparts a stronger therapeutic response and suppression of metastasis by virtue of sustained CpG release and signaling within the tumor.

Overall, our findings demonstrate the importance of optimizing the release kinetics of immunostimulatory agents such as CpG for cancer therapy. While we only investigated the ability to tune CpG release using our system, it is important to note that many other immunostimulatory agents are negatively‐charged (such as poly I:C or other nucleic acids),^[^ ^37^ ^]^ meaning that—as we demonstrate with ELP‐CpG—this system could be employed to deliver those agents and study the effects of tuning their release kinetics. We have shown that by optimizing the formulation of a single adjuvant therapy, we can achieve significant and clinically relevant effects on antitumor immunity and metastatic disease.

## 4 Conclusion

As interest in immunotherapy and controlled‐release biomaterials surges, researchers continue to explore the effects of tuning the release and exposure of immunotherapies on the systemic immune response. In this study, we have shown the importance of optimizing the release kinetics of CpG using a precisely tunable, depot‐forming ELP mixture. Altering the release profile of CpG significantly affects cumulative CpG exposure, reduces the incidence of metastatic disease, and shifts the local and systemic immune landscape toward a more immunogenic state. In conclusion, we show that this platform can be used to optimize the delivery of immunotherapies and establish the groundwork for the use of ELP depots to improve treatment outcomes in immunotherapy.

## 5 Experimental Section

### ELP Cloning, Expression, and Purification

The ELP genes were assembled in a modified pET‐24a^+^ vector using plasmid reconstruction by recursive directional ligation (PRe‐RDL).^[^ ^38^ ^]^ Briefly, the dsDNA (Table S1, Supporting Information) encoding the Lys_12_ domain was purchased (Integrated DNA Technologies, Coralville, IA) and integrated into the vector using Gibson Assembly Master Mix (New England Biolabs, Ipswich, ME). The Lys_12_‐containing vector was then digested with BseRI and BglI, a similar vector containing the ELP with a [Val‐Pro‐Gly‐Val‐Gly]_60_ sequence was digested with AcuI and BglI (New England Biolabs, Ipswich, ME), and the two digestion products were ligated together to create a vector encoding the ELP with a C‐terminal Lys_12_ tag, the sequence of which is available in the Table S2 (Supporting Information). Similar ELPs containing oligolysine tags of four and eight residues (Lys_4_ and Lys_8_) were also cloned, as described above. The sequence of the excipient ELP used in this study is 60 repeats of the same ELP sequence as in ELP‐Lys_12_ but missing the Lys_12_ sequence shown in Table S2 (Supporting Information).

The ELPs were expressed by transforming the pET‐24a^+^ vectors into competent BL21(DE3) *Escherichia Coli* (*E. coli*, New England Biolabs, Ipswich, ME). The cultures were grown in shake flasks containing 2xYT broth (Fisher Scientific, Fair Lawn, NJ) with kanamycin sulfate (Millipore Sigma, St. Louis, MO) at 37 °C and at 200 rpm for 18 h. The cultures were then centrifuged, resuspended in cold 1X phosphate‐buffered saline (PBS), and sonicated to lyse the cells (Q500 sonicator, QSonica, Newtown, CT). Polyethyleneimine (PEI, MP Biomedicals, Irvine, CA) was added to precipitate nucleic acids, and the lysate was clarified by centrifugation at 4 °C at ≈27000 x g for 10 min. Inverse transition cycling (ITC), a technique was developed to purify ELPs and their fusions,^[^ ^16a^ ^]^ was performed for 2–3 cycles to purify the ELP from other bacterial contaminants. For ELP‐Lys_n_ fusions, complexed contaminant nucleic acids were removed using fast performance liquid chromatography (FPLC, GE Healthcare, Chicago, IL) and an anion exchange resin column (HiTrap Q HP, Cytiva, Uppsala, Sweden). The ITC‐ and anion exchange‐purified ELP was then dialyzed into Milli‐Q water to remove residual salts. Bacterial endotoxin was removed using Mustang E membrane syringe‐driven filters (Pall Corporation, New York, NY) to an endotoxin level of less than 0.038 EU mg^−1^. The purified protein was finally characterized by SDS‐PAGE (Figure S5, Supporting Information, Bio‐Rad, Hercules, CA) and the molecular weight of the ELPs was confirmed by matrix assisted laser desorption ionization time of flight mass spectrometry (MALDI‐TOF MS) (Figure S6, Supporting Information).

### Binding of CpG to ELP‐Lys_n_ and Phase Behavior of ELP‐CpG Complex

The binding of ELP‐Lys_12_ to CpG ODN 1826 (AdipoGen Corporation, San Diego, CA) was characterized by a DNA gel‐shift assay. Solutions containing either CpG only (9.99 µм), CpG with excipient ELP, or the CpG‐binding ELP formulations were mixed (33.3 µм total ELP concentration) in PBS on ice and run on a 1% (w/v) agarose gel (Table 1). Retardation of the CpG in the gel was indicative of electrostatic complexation with the ELP. The thermal phase transition behavior of the ELP‐CpG mixtures was characterized by temperature‐programmed UV–vis spectrophotometry. A Cary 300 and Cary 3500 UV–vis spectrophotometer (Agilent Technologies, Santa Clara, CA) were used to measure the optical density at 350 nm of ELP‐CpG formulations in PBS at 1044, 522, 261, 130.5, 65.25, 32.625, and 16.3125 µм from 15 °C to 40 °C. Samples were heated and cooled at a rate of 0.66 °C min^−1^, and the optical density was recorded every 0.33 °C increment. The ELP T_t_ was defined as the temperature with the greatest slope in the optical density versus temperature plot.

### Surface Plasmon Resonance (SPR)

SPR experiments were performed on a Biacore T200 instrument (Cytiva, Uppsala, Sweden) with a Series S PEG chip. CpG ODN 1826 labeled with biotin at the 3′ terminus (InvivoGen, San Diego, CA) was used for immobilization to the SPR chip, as follows. First, NeutrAvidin (Thermo Fisher Scientific, Waltham, MA) at 10 µg mL^−1^ in 10 mм sodium acetate (pH 5.0) was immobilized via standard EDC/NHS amine coupling at 5 µL min^−1^ until the signal saturated. The surface was then quenched with an injection of 1 м ethanolamine‐HCl (pH 8.5) for 420 s. The surface was then functionalized by capturing CpG‐biotin (10 µg mL^−1^) for 1200 s to achieve complete saturation. Analyte binding was assessed at 30 µL min^−1^, with a 120 s association and a 600 s dissociation phase with a concentration range of 3.125‐100 µм of ELP. All experiments were conducted at 25 °C with PBS + 0.005% (v/v) Surfactant P‐20 as running buffer. Binding was evaluated using the BiacoreT200 Evaluation software using a steady state affinity model due to significant interactions between the analytes and the reference channel that precluded analysis by a kinetic model.

### Bioactivity of ELP‐CpG

The bioactivity of ELP‐CpG was quantified by a RAW264.7 nitrite assay (Figure 1C).^[^ ^17b^ ^]^ Briefly, RAW264.7 cells (American Type Culture Collection, Manassas, VA) were cultured in DMEM (Gibco, Thermo Fisher Scientific, Waltham, MA) with 10% FBS at 37 °C and 5% CO_2_. To evaluate bioactivity of CpG‐bound ELP, 1 × 10^6^ cells were incubated for 24 h with either: PBS, ELP‐Lys_12_ alone, 5 µg mL^−1^ of soluble CpG, or ELP‐Lys_12_ complexed with 5 µg mL^−1^ CpG at a 1:1 N:P ratio. Subsequently, cell supernatants were collected, and nitrite concentration was quantified using a Griess reagent assay (Thermo Fisher Scientific, Waltham, MA).

### Microscopic Characterization of ELP‐CpG Depot Morphology and Self‐Assembly

ELP‐Lys_12_ was fluorescently labeled at the N‐terminus with Alexa Fluor 594 using an NHS ester labeling kit (Thermo Fisher Scientific, Waltham, MA). Similarly, a commercially available version of CpG ODN 1826 labeled at its 3′‐end with fluorescein isothiocyanate (FITC) was obtained (InvivoGen, San Diego, CA). Formulations (Table 1) containing 1044 µм total ELP concentration—with 2% (v/v) of AF594‐ELP‐Lys_12_ and 1% (v/v) of FITC‐CpG—were mixed on ice, transferred to a glass microscope slide, and imaged using a Zeiss Axio Imager.D2m with a sample stage pre‐heated to 37 °C (Carl Zeiss, Jena, Germany). Samples were imaged (Figure S10, Supporting Information) after heating for ≈5 min. Confocal laser scanning microscopy was also used to characterize the ELP‐CpG coacervate morphology. Samples were prepared identically as above—apart from a 1% (v/v) ratio of AF594‐ELP‐Lys_12_ used—and transferred to a 384‐well #1.5 glass‐bottom plate, heated to 37 °C, and immediately imaged over time as the ELP phase transition occurred using a Zeiss 710 inverted confocal microscope (Carl Zeiss, Jena, Germany) at the Duke University Light Microscopy Core Facility. Fluorescence recovery after photobleaching (FRAP) was performed using a 488 nm argon laser for 2 s to bleach an area of ≈28 µm^2^. To account for photofading, intensity values of the bleached regions at each time point were normalized to the lost fraction of intensity of an unbleached region in the same field.

### Small‐Angle X‐Ray Scattering (SAXS)

SAXS data were collected at the 16ID‐LiX Beamline of the National Synchrotron Light Source II (NSLS‐II, Brookhaven National Laboratory, Upton, NY). The X‐ray wavelength was set to 0.8189 Å and both small‐ and wide‐angle X‐ray scattering (SAXS and WAXS) setups were utilized simultaneously to cover scattering ranges of 0.006 ≤ q ≤ 3.19 Å^−1^, where q = (4π/λ)sinθ, with 2θ representing the scattering angle and λ indicating the X‐ray wavelength. For room temperature measurements, the sample solution and matching buffer were measured in a capillary flow cell. Data frames (10–16) were recorded with a 0.5 s exposure time per frame while the sample flowed in a single direction. For temperature‐dependent measurements at 37 °C, custom‐designed flat fixed cells with mica windows and temperature control capabilities were employed. Before each solution scattering measurement, both the empty cells and matching buffer were measured for proper background subtraction. To minimize radiation damage, twenty repeated measurements were performed at different positions on the sample, with each frame taken at a 0.5 s exposure time.

The 2D scattering images collected by the SAXS and WAXS detectors were converted into 1D scattering profiles and merged. Transmission corrections and background subtraction were applied to minimize the water peak at ≈2.0 Å^−1^. The final scattering profiles were obtained by averaging the repeated measurements after eliminating outliers and subtracting contributions from the empty cell and matching buffer. Data processing was performed using LiXTools (https://github.com/NSLS‐II‐LIX/lixtools) and the Python package py4xs (https://github.com/NSLS‐II‐LIX/py4xs) in NSLS‐II Jupyter Notebook environment.^[^ ^39^ ^]^ The final subtracted 1D curves were further analyzed using the Irena package for analysis of small‐angle scattering data.^[^ ^40^ ^]^

### Establishing Orthotopic Mouse Model of Breast Cancer

All in vivo studies were performed with approval by the Duke University Institutional Animal Care and Use Committee (IACUC) under protocols A237‐20‐12 and A242‐23‐12. 4T1 murine mammary carcinoma cells (American Type Culture Collection, Manassas, VA) were cultured in RPMI‐1640 media (ATCC modification, Thermo Fisher Scientific, Waltham, MA) with 10% FBS at 37 °C and 5% CO_2_. On the day of inoculation, cells were trypsinized, centrifuged, and resuspended in serum‐free RPMI‐1640 media at a concentration of 1 × 10^6^ cells mL^−1^. Female 6–8 week old BALB/cJ mice (The Jackson Laboratory, Bar Harbor, ME) were purchased and housed at either the Duke University Cancer Center Isolation Facility or Genome Sciences Research Building II. Mice were shaved around the abdomen and anesthetized with 2.0% isoflurane vapor and inoculated with 0.5–1 × 10^5^ 4T1 cells in the 4^th^ mammary fat pad. Tumors were grown to a volume of ≈100 mm^3^ prior to treatment, and the volume of each tumor was determined by the formula:

(3) $$\mathrm{volume}\;({\mathrm{mm}}^{3})=\mathrm{leng}\mathrm{th}\;(\mathrm{mm})\;x\;\mathrm{wid}\mathrm{th}\;(\mathrm{mm})\;x\;0.52$$

### Characterizing Intratumoral CpG Retention

Alexa Fluor 647‐labeled ODN 1826 (AF647‐CpG) was purchased (Integrated DNA Technologies, Coralville, IA) and mixed at a 2% (v/v) ratio with unlabeled CpG prior to mixing with ELP. To track intratumoral retention of CpG in vivo, female BALB/cJ mice bearing orthotopic ≈100 mm^3^ 4T1 tumors were injected with the ELP‐CpG formulations described in Table 1, soluble CpG, or PBS to measure background fluorescence. Fifty microliters of ELP‐CpG or soluble CpG were injected into the core of the tumors through a 27½ gauge needle attached to a 1 mL syringe. After injection, mice were imaged using an IVIS Lumina III in vivo imaging system (Revvity, Waltham, MA). Images were analyzed using Living Image 4.8.2 software (Revvity, Waltham, MA), with average radiant efficiency being calculated within a single region of interest drawn around the injected site for each mouse immediately after injection.

### Evaluation of In Vivo Efficacy

Female 6–8 week old BALB/cJ mice were inoculated with orthotopic 4T1 tumors, as described previously. Once tumors grew to ≈100 mm^3^ on day 0, mice were injected *i.t*. with 50 µL of either PBS, soluble CpG (2 µg µL^−1^), or 1044 µм ELP‐CpG in either the 100, 80, 60, or 40 formulations as described in Table 1 using a 27½ gauge needle that was kept on ice and actuated using a syringe pump at an injection rate of 120 µL min^−1^. Body weight of mice was measured using a digital scale. Tumor size was continuously measured, and mice were sacrificed when they either exhibited >15% body weight loss, their tumor volume exceeded 1650 mm^3^, or they exhibited signs of severe lack of mobility or breathing difficulty. To quantify lung metastases, mice were euthanized and the abdominal cavity was opened to reach the lungs. A solution of 15% India ink was injected into the lungs through the trachea. Following this, the lungs were resected and washed in water, fixed using Fekete's solution, and lung metastases were visually counted and their area was calculated using Fiji/ImageJ software (National Institutes of Health, Bethesda, MD) by digitally outlining the area of high‐resolution lung images, subsequently outlining the area of individual metastases, and calculating the fractional area of lung metastases relative to lung area. Lung pairs which were not fully perfused with staining solution or resected intact (i.e., only one lung was harvested or a lobe was lost) were excluded from analysis.

Immunotyping was performed by excising the tumors and spleens from euthanized mice following lung resection and preserved in PBS on ice. Spleens were homogenized in media (RPMI‐1640) through a 70 µm cell strainer using the plunger of a 5 mL syringe. The resulting cell suspension was collected and centrifuged at 1200 rpm, ACK lysing buffer was added, and the red blood cells were lysed for 5 min at room temperature. The suspension was then centrifuged, the pellet was washed with RPMI‐1640 media, and resuspended. The washed suspension was overlaid onto Lympholyte‐M (Cedarlane Labs, Ontario, Canada), centrifuged according to the manufacturer's instructions, and the clear lymphocyte layer was collected from the resulting gradient via pipetting. Lymphocytes were finally washed and resuspended in flow cytometry buffer (5% FBS in PBS). Tumors were similarly processed into a single cell suspension.^[^ ^41^ ^]^ Briefly, tumors were digested in 0.1% collagenase type I, 0.2% dispase type I, and 1% DNAse I at 37 °C for 30 min, strained through a 70 µm cell strainer, washed with flow buffer, and centrifuged at 350 rcf. Supernatants were collected and analyzed according to manufacturers’ instructions using ELISAs for CpG‐ODN (MyBioSource, San Diego, CA) and mouse IFNγ (Thermo Fisher Scientific, Waltham, MA). ACK lysis buffer was added as described previously and the resulting suspension was passed further through a 40 µm cell strainer. The collected cell suspension was centrifuged, washed with flow buffer, and finally collected for analysis.

Flow cytometry analysis was performed by first preparing a master mix of antibodies used for staining. Antibodies (PerCP‐αCD45, PE/cy7‐αCD11c, FITC‐αCD86, and eFluor 450‐αMHC‐II for the first experiment, and FITC‐αCD45, APC‐αCD11c, PE/Cy7‐αCD86, APC‐eFluor 780‐αMHC‐II, Brilliant Violet 510‐αCD8α, and Pacific Blue‐αF4/80 for the second experiment) were diluted in flow buffer according to manufacturer recommendations (1:160 dilution). Rat Anti‐Mouse CD16/CD32 Fc Block was added and incubated with cell suspensions on ice for 10 min, after which the antibody cocktail was added to each sample. Samples were incubated with antibodies on ice for 30 min, after which they were washed/centrifuged with flow buffer, stained with propidium iodide (PI) live/dead stain, and kept on ice until analysis. Antibody compensation beads (Thermo Fisher Scientific, Waltham, MA) were prepared similarly to the sample cells, and a live/dead cell control was prepared by heat‐killing cells at 65 °C for 5 min and then adding the dead cells back to an equal volume of live cells prior to staining with PI solution. Samples were analyzed using a BD FACSCanto II flow cytometer (Beckton Dickinson, Franklin Lakes, NJ) in the Duke Cancer Institute Flow Cytometry Core Facility. Exported FCS files were analyzed using FlowJo 10 software (Beckton Dickinson, Franklin Lakes, NJ).

### Statistical Analysis

Statistical analyses were performed in GraphPad Prism 10 software, which was also used to generate figures for the manuscript. All data were presented as mean values ± standard error of the mean (SEM). Cell assay data, ELP morphometric data, and analyses of groups of experimental replicates were analyzed using ANOVA with Tukey's post‐hoc multiple comparisons test. Flow cytometry data were analyzed using ANOVA with Tukey's post‐hoc test after checking normality of data by multiple tests (including D'Agostino & Pearson, Anderson‐Darling, Shapiro‐Wilk, and Kolmogorov‐Smirnov). Tumor growth data were analyzed using repeated‐measures ANOVA and survival was analyzed using Log‐rank/Mantel‐Cox tests. In all statistical tests, significance was defined as *p*<0.05 and denoted on figures with an asterisk (*) unless otherwise noted in the caption.

## Conflict of Interest

The authors declare no conflict of interest.

## Supporting information



Supporting Information

## Data Availability

The data that support the findings of this study are available from the corresponding author upon reasonable request.
