# Supplementary material for: Controlling Release Kinetics of an Adjuvant from a Depot Improves the Efficacy of Local Immunotherapy in Metastatic Cancer
Source: Adv Sci (Weinh). 2025 Jul 6;12(37):e03591. doi: 10.1002/advs.202503591 (PMC12499502; doi:10.1002/advs.202503591)
Supplement: Supplementary file 1 — Supporting Information [file ADVS-12-e03591-s001.docx]

**Supplemental Information**

**Controlling release kinetics of an adjuvant from a depot improves the efficacy of local immunotherapy in metastatic cancer**

Joshua J. Milligan^1^, Rachel L. Strader^1^, Arunangshu Chakrabarty^1^, Max Ney^1^, Parul Sirohi^1^, Jonathan C. Su^1^, Anastasia K. Varanko^1^, Yulia Shmidov^1^, Kalina Tsolova^2^, Cassio Mendes Fontes^1^, Anna Finkelstein^1^, Rohinee Mattikalli^1^, Yun-Xing Wang^3^, Lixin Fan^4^, Xinghai Li^1^, Smita K. Nair^5^, and Ashutosh Chilkoti^1*^

^1^Department of Biomedical Engineering, Duke University, Durham, NC 27708

^2^Department of Biochemistry, Duke University School of Medicine, Durham, NC 27708

^3^Center for Structural Biology, Center for Cancer Research, National Cancer Institute, Fredrick, MD 21702

^4^Basic Science Program, Fredrick National Laboratory for Cancer Research, SAXS Facility of the National Cancer Institute, Fredrick, MD 21702

^5^Department of Surgery, Duke University Medical Center, Durham, NC 27708

*corresponding author

**Figure S1: SDS-PAGE characterization of ELP-Lys_4_ and Lys_8_.** First lane: molecular weight ladder (Bio-Rad Precision Plus), second and third lanes: ITC-purified ELP-Lys_4_ and ELP-Lys_8_, respectively ([VPGVG]_60_[K]_4 or 8_GWP).

**Figure S2: ELP-Lys_4_ and -Lys_8_ exhibit reversible, thermally-responsive phase behavior without (A) and with (B) complexed CpG.** Samples (1000 µM) were heated in a UV-vis spectrophotometer from 15 °C to 55 °C (solid lines), and then immediately cooled back to 15 °C (dashed lines). Absorbance at 350 nm correlates with the optical turbidity of the sample.

**Figure S3:** **Binding affinity of CpG to ELP-Lys_n_ is tunable by changing the number of Lys residues within the ELP fusion.** **(A)** ELP (33 µM) was mixed with CpG at a 1:1 N:P ratio for each ELP-Lys_n_ construct, run in an agarose gel stained with SYBR-Safe DNA stain, and imaged with a UV transilluminator. Lanes (left-to-right) consist of: 10 KB DNA ladder, CpG-only control, ELP-Lys_4_, ELP-Lys_8_, and ELP-Lys_12_. An increased fraction of CpG (20 bp) migrating towards the bottom of the gel is indicative of less ELP-CpG complexation. **(B)** Raw SPR curves (response units (RUs) vs. time (s)) for ELP-Lys_4, 8, 12_ binding to immobilized CpG-biotin. **(C)** Steady-state normalized affinity plots for ELP-Lys_n_ constructs measured in Figure S3B. **(D)** K_D_ and Chi^2^ values from fitted, normalized SPR data.

**Figure S4:** **Altering CpG binding affinity by changing the number of Lys residues within the ELP fusion does not affect treatment efficacy in combination with radiotherapy.** Female BALB/c mice (n=6-8) were inoculated with orthotopic 4T1 mammary carcinoma tumors. Upon growth of the tumor to approx. 100 mm^3^ in volume, mice were treated *i.t.* with 100 µg of CpG complexed to ELP-Lys_4_, -Lys_8_, or -Lys_12_ at a 1:1 N:P ratio. 24 h later, mice were treated *i.t.* with 3.3 µCi/mm^3^ tumor of ^131^I-ELP brachytherapy as described in [14]. **(A)** Tumor volume over time. **(B)** Kaplan-Meier survival analysis. Error bars: 1 standard error of the mean.

**Figure S5: SDS-PAGE characterization of excipient ELP and ELP-Lys_12_.** First lane: molecular weight ladder (Bio-Rad Precision Plus), second lane: ITC-purified excipient ELP ([VPGVG]_60_), third lane: ITC- and anion exchange-purified ELP-Lys_12_ ([VPGVG]_60_[K]_12_GWP).

**Figure S6: MALDI-TOF characterization of ELP-Lys_12_ and excipient ELP.** **(A)** Mass spectrogram denoting 26.526 kDa peak (m/z) of ELP-Lys_12_. **(B)** Mass spectrogram denoting 24.630 kDa peak (m/z) of excipient ELP. Samples were diluted 1:10 in sinapinic acid (SA) matrix and 1 µL droplets were applied onto an MTP AnchorChip 384-well polished steel target plate. Samples were air-dried and loaded into a Bruker Autoflex Speed LRF MALDI-TOF mass spectrometer. A linear mode, negative polarity method scanning from 10 kDa to 40 kDa was used to collect data for all samples, and the system calibration was verified using a ProteoMass aldolase MALDI-MS standard (39.21128 kDa).

**Figure S7: ELP-CpG exhibits reversible, thermally-responsive phase behavior.** Samples (comprising 100, 80, 60, and 40 formulations as described) at seven dilutions were heated in a UV-vis spectrophotometer from 15 °C to 40 °C (solid lines), and then immediately cooled back to 15 °C (dashed lines). Absorbance at 350 nm correlates with the optical turbidity of the sample.

**Figure S8: ELP-CpG and excipient ELP alone reversible, thermally-responsive phase behavior.** Samples at 522 µM, and diluted to 65.25 µM, were heated in a UV-vis spectrophotometer from 8 °C to 40 °C (solid lines), and then immediately cooled back to 8 °C (dashed lines). Absorbance at 350 nm correlates with the optical turbidity of the sample.

**Figure S9: ELP-Lys_12_ mixed with excipient ELP and CpG-ODN 1826 undergoes liquid-liquid phase separation into droplets upon heating.** Fluorescence microscopy images of ELP-containing mixtures (1044 µM total ELP concentration) with varying ratios of ELP-Lys_12_ and excipient ELP. ELP was mixed with CpG (313 µM), a drop placed on a glass slide, heated to 37 °C, and imaged using a fluorescence microscope. 2% (v/v) of ELP-Lys_12_ is labeled with Alexa Fluor 594 dye (yellow in above image) and 1% of CpG is labeled with FITC dye (green in above image).

**Figure S10: ELP-Lys_12_ mixed with CpG does not exhibit liquid-like behavior after heating and settling.** **(A)** Fluorescence recovery after photobleaching (FRAP) data normalized to background and minimum fluorescence values in images. **(B)** Representative images from ELP-CpG 100 sample showing initial state at t=0s, bleached spots (denoted by white arrows) immediately after photobleaching at t=2s, and after minimal recovery at t=20s. Samples (n=3 spots bleached per formulation) were heated for 10 minutes at 37 °C prior to imaging. Error bars: 1 standard error of the mean.


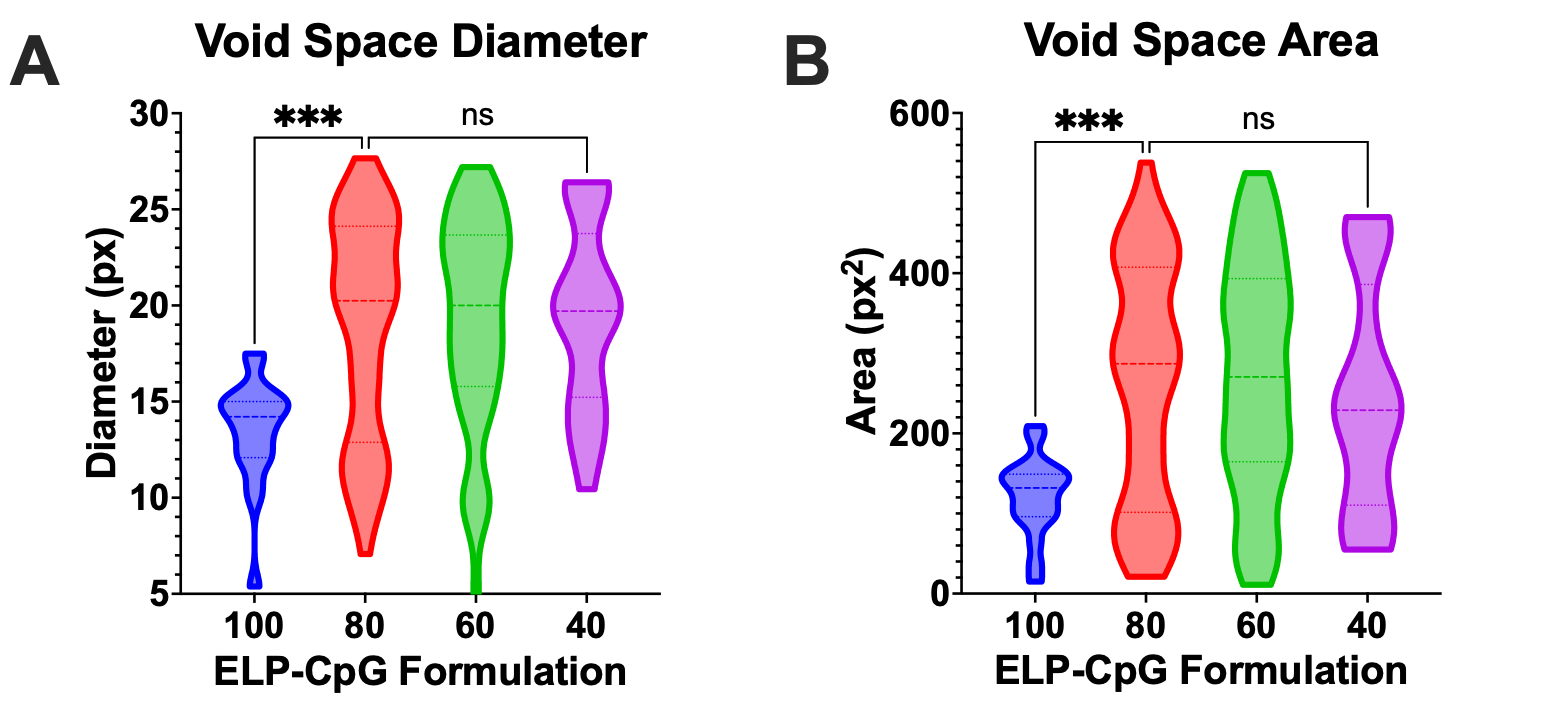


**Figure S11: Morphometric analysis of ELP-CpG void space diameter and area.** **(A)** Diameter and **(B)** area of void spaces sampled from images in **Figure 2C**. Analysis was performed using FIJI/ImageJ software by loading images of FITC-CpG channel from each sample, converting to an 8-bit image, thresholding and converting image to a binary map, and analyzing hole diameter and area. ns: not significant, ***p<0.001 (ANOVA, Tukey).

**Figure S12: SAXS spectra (intensity vs. scattering vector q) for ELP-Lys_12_, excipient ELP, and CpG samples. (A)** Excipient ELP at room temperature at three concentrations (522 µM – top, 261 µM – middle, and 130.5 µM – bottom). Curves are offset for clarity. **(B)** ELP-Lys_12_ at room temperature at same concentrations as excipient ELP sample. **(C)** CpG at room temperature at three concentrations (313.2 µM – top, 156.6 µM – middle, and 78.3 µM – bottom). **(D)** ELP-Lys_12_ (522 µM) mixed with CpG (313.2 µM) at a 1:1 N:P ratio at room temperature. Black curve represents the theoretical scattering curve assuming no interactions, produced by mathematical addition of the CpG scattering curve to the ELP-Lys_12_ scattering curve. Brown line represents the measured scattering curve. Differences between theoretical and measured curves indicate conformational changes driven by interactions between ELP-Lys_12_ and CpG. **(E)** ELP-Lys_12_ (522 µM), excipient ELP (522 µM), and CpG (313.2 µM) comprising the ELP-CpG 100 formulation at room temperature (top curve). Three total ELP concentrations (1044 µM – top, 522 µM – middle, and 261 µM – bottom) are measured, with CpG diluted accordingly to maintain a 1:1 N:P ratio with ELP-Lys_12_. **(F)** Identical ELP-CpG 100 samples heated to 37 °C.

**Figure S13: Mixtures of ELP-Lys_12_ with excipient ELP and CpG phase-separate into ELP-poor “void spaces” throughout depots that are rich in soluble CpG.** Confocal microscopy composite images of seven formulations comprising AF594-labeled ELP-Lys_12_, FITC-labeled CpG, and AF405-excipient ELP. Scale bar: 20 µm.

**Fig. S14:** **ELP-CpG nanocomplex prolongs retention of CpG within tumors.** IVIS *in vivo* representative images of AF647-CpG epi-fluorescence in female BALB/c mice injected *i.t.* with 2% (v/v) CpG either soluble or in complex with ELP (40, 60, 80, and 100 formulations).


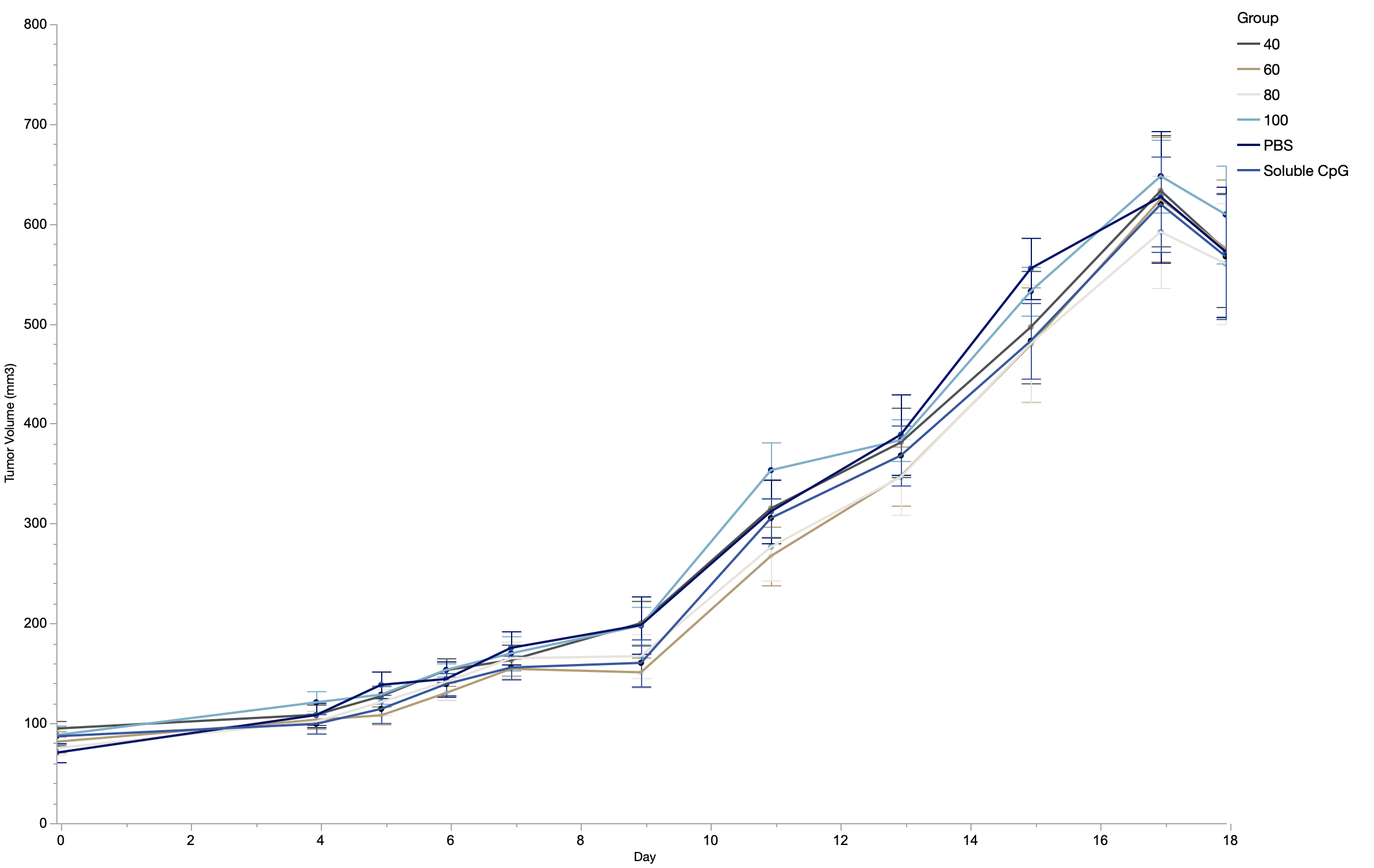


**Figure S15: ELP-CpG therapy alone does not significantly affect tumor growth in mice.** Female BALB/c mice (n=8) were inoculated with orthotopic 4T1 mammary carcinoma tumors. Upon growth of the tumor to approx. 100 mm^3^ in volume, mice were treated *i.t.* with ELP-CpG in either the 100, 80, 60, or 40 formulations, soluble CpG, or PBS. All CpG-containing formulations comprised 100 µg of CpG. Error bars: 1 standard error of the mean (SEM).

**Figure S16: ELP-CpG monotherapy does not significantly increase survival in mice with 4T1 mammary carcinoma.** Kaplan-Meier survival plot of treated mice (n=8) shown in Fig. S3. Median survival for groups was calculated as follows (in days): 100: 24, 80: 27, 60: 24, 40: 25, Soluble CpG: 24.5, PBS: 25.5.

**Figure S17: CD45^+^ cell count, frequency, and expression inside the tumor in treated mice.** Flow cytometry data summarizing **(Left)** number of intratumoral CD45^+^ cells (among live cells), **(Middle)** CD45^+^ cell frequency, and **(Right)** CD45 expression among as measured by mean fluorescent intensity (MFI, geometric mean). Error bars represent ± one standard error from the mean (SEM).

**Figure S18: Changes in CD11c expression.** Flow cytometry data (n=7-8) summarizing **(A)** number of CD11c^+^ cells (among CD45^+^ cells), **(B)** CD11c^+^ cell frequency (as a percentage of CD45^+^ cells), and **(C)** CD11c expression among CD11c^+^ cells as measured by mean fluorescent intensity (MFI, geometric mean). Error bars represent ± one standard error from the mean (SEM).

**Figure S19: MHC class II^+^ cell frequency and expression. (A)** MHC class II^+^ cell frequency as a percentage of CD11c^+^ cells. **(B)** Mean fluorescent intensity (MFI, geometric mean) of MHC class II expression among positive cells. Error bars represent ± one standard error from the mean (SEM).

**Figure S20: CD45^+^ cell count, frequency, and expression within the spleens of treated mice.** Flow cytometry data summarizing **(Left)** number of splenic CD45^+^ cells (among live cells), **(Middle)** CD45^+^ cell frequency, and **(Right)** CD45 expression among as measured by mean fluorescent intensity (MFI, geometric mean). Error bars represent ± one standard error from the mean (SEM).

**Figure S21: CD11c expression, CD86^+^ cell frequency, and MHC class II cell frequency. (A)** Mean fluorescent intensity (MFI, geometric mean) of CD11c expression levels in isolated splenocytes. **(B)** CD86^+^ cell frequency as a proportion of CD11c^+^ cells. **(C)** MHC class II^+^ cell frequency as a proportion of CD86^+^ cells. Error bars represent ± one standard error from the mean (SEM).

**Figure S22: *i.t.* CpG and IFNγ levels from samples 3 d following treatment.** (**A**) Weight-normalized CpG levels and (**B**) IFNγ levels from excised tumor homogenates (PBS: blue, soluble CpG: red, ELP-CpG: green). Error bars: one standard error from the mean (SEM). ns: not significant, *p<0.05 (ANOVA, Tukey).

**Figure S23: Representative flow cytometry dot plots.** Top row: CD45^+^CD8^+^ T cells, second row: CD11c^+^MHC-II^+^ DCs, third row: CD86^+^MHC-II^+^ DCs, bottom row: CD45^+^F4/80^+^ macrophages within each experimental group (left column: PBS, middle column: soluble CpG, right column: ELP-CpG) from tumor samples harvested 3 d following treatment.

**Figure S24: DC populations and DC activation states within tumors 3 d following treatment.** (**A**) Weight-normalized number of CD11c^+^ DCs within tumors for experimental groups (PBS: blue, soluble CpG: red, ELP-CpG: green). (**B**) Weight-normalized number of CD11c^+^CD86^+^ activated DCs within each tumor sample. Error bars: one standard error from the mean (SEM). ns: not significant (ANOVA, Tukey).

| **Description** | **Sequence (5’ 🡪 3’)** |
| --- | --- |
| Lys_12_ DNA fragment | TCTAGAAATAATTTTGTTTAACTTTAAGAAGGAGGAGTACATATGGGCAAGAAAAAGAAGAAAAAGAAGAAGAAGAAAAAGAAGGGCTGGCCGTAATGATCTTCAGGATCCGAATTCGAGCTCCGTC |

**Table S1:** Gene sequences used for cloning Lys_12_ tag into ELP-containing plasmid.

| **ELP** | **Amino Acid Sequence** | **Molecular Weight (kDa)** |
| --- | --- | --- |
| ELP-Lys_12_ | GVGVPGVGVPGVGVPGVGVPGVGVPGVGVPGVGVPGVGVPGVGVPGVGVPGVGVPGVGVPGVGVPGVGVPGVGVPGVGVPGVGVPGVGVPGVGVPGVGVPGVGVPGVGVPGVGVPGVGVPGVGVPGVGVPGVGVPGVGVPGVGVPGVGVPGVGVPGVGVPGVGVPGVGVPGVGVPGVGVPGVGVPGVGVPGVGVPGVGVPGVGVPGVGVPGVGVPGVGVPGVGVPGVGVPGVGVPGVGVPGVGVPGVGVPGVGVPGVGVPGVGVPGVGVPGVGVPGVGVPGVGVPGVGVPGVGVPGVGVPGKKKKKKKKKKKKGWP | 26.523 |
| Excipient ELP | GVGVPGVGVPGVGVPGVGVPGVGVPGVGVPGVGVPGVGVPGVGVPGVGVPGVGVPGVGVPGVGVPGVGVPGVGVPGVGVPGVGVPGVGVPGVGVPGVGVPGVGVPGVGVPGVGVPGVGVPGVGVPGVGVPGVGVPGVGVPGVGVPGVGVPGVGVPGVGVPGVGVPGVGVPGVGVPGVGVPGVGVPGVGVPGVGVPGVGVPGVGVPGVGVPGVGVPGVGVPGVGVPGVGVPGVGVPGVGVPGVGVPGVGVPGVGVPGVGVPGVGVPGVGVPGVGVPGVGVPGVGVPGVGVPGVGVPGVGVPG | 24.644 |

**Table S2:** Amino acid sequences of ELPs. ELP-Lys_12_ sequence contains a C-terminal Trp residue to aid in calculation of nucleic acid contamination via UV-vis spectrophotometry (A_260_/A_280_).
